# Gender Differences in Intimate Partner Violence Victimization and Its Relationships With Anxiety, Depression Symptoms and Suicide Behaviours in China

**DOI:** 10.3389/ijph.2025.1607953

**Published:** 2025-02-18

**Authors:** Li Lu, Lizhen Ye, TianTian Zhang, Rong Li, Chang Chen, Zixuan Cao, Bing-Cun Ma, Zhan-Cui Dang, Baeksan Yu, Ole A. Andreassen, Qing Shen, Zhongliang Zhou, Sha Lai, Shou Liu

**Affiliations:** ^1^ Institute of Health Management and Policy, School of Public Policy and Administration, Xi’an Jiaotong University, Xi’an, China; ^2^ System Behavior and Management Laboratory, Philosophy and Social Sciences Laboratory of the Ministry of Education, Xi’an Jiaotong University, Xi’an, China; ^3^ Department of Public Health, Erasmus MC—University Medical Center Rotterdam, Rotterdam, Netherlands; ^4^ Department of Nosocomial Infection Management, Xi’an Central Hospital, College of Medicine, Xi’an Jiaotong University, Xi’an, China; ^5^ MSD R&D (China) Co., Ltd., Beijing, China; ^6^ Department of Public Health, Medical College, Qinghai University, Xining, China; ^7^ Department of Education, Gwangju National University of Education, Gwangju, Republic of Korea; ^8^ NORMENT Centre, Division of Mental Health and Addiction, University of Oslo and Oslo University Hospital, Oslo, Norway; ^9^ Clinical Research Center for Mental Disorders, Shanghai Pudong New Area Mental Health Center, Tongji University School of Medicine, Shanghai, China; ^10^ Institute for Advanced Study, Tongji University, Shanghai, China

**Keywords:** intimate partner violence victimization, gender difference, anxiety symptoms, depressive symptoms, suicide ideation, suicide attempt

## Abstract

**Objectives:**

To investigate the gender difference in Intimate partner violence (IPV) victimization and its association with mental health, examine social-demographic and health characteristics-specific relationships.

**Methods:**

This cross-sectional study evaluated lifetime prevalence of total, psychological, physical and sexual IPV victimization. Gender-stratified multiple logistic regressions were performed to examine associations between total and subtypes of IPV victimization and anxiety and depressive symptoms, suicide ideation and suicide attempt. Sensitivity analyses and stratification analyses were additionally conducted.

**Results:**

Among 21,824 participants (female: 44.7%), females reported higher total, psychological and physical but not sexual lifetime prevalence of IPV victimization than males. Specifically, male participants with psychological (OR = 3.62, 95% CI: 2.58–5.08 vs. OR = 1.87, 95% CI: 1.39–2.51) or sexual (OR = 4.02, 95% CI: 2.61–6.20 vs. OR = 1.46, 95% CI: 0.91–2.35) IPV victimization presented greater odds of presenting possible anxiety than females; males with physical IPV victimization showed greater likelihood of with suicide ideation than females (OR = 9.95, 95% CI: 6.68–14.82 vs. OR = 4.61, 95% CI: 3.02–6.15).

**Conclusion:**

Prevention programs should be tailored to respond to IPV in various contexts to reduce the likelihood of and the detrimental effects of IPV.

## Introduction

Intimate partner violence (IPV) has emerged as a critical and debilitating global health concern that leads to physical, sexual or psychological harm [1], globally, 27% of ever-partnered women aged 15–49 years are reported to have experienced physical, sexual, or both, IPV in their lifetime [2]. IPV has been further exacerbated by social isolation during the COVID-19 pandemic [3], since substantial self-quarantine at home can led to constant contact between victims and perpetrators, victims of IPV are unable to safely connect with services (such as domestic-violence hotlines), resulting in increased violence and decreased reports and thus worsen already tenuous situations [3, 4]. One review found an increase in domestic violence cases during the pandemic, especially during the first week of the COVID-19 lockdown in each countries, and the eligible studies from the Asia-Pacific region only came from Australia and New Zealand [3]; one included study reported that 15.9% of the frontline workers and service providers indicated the first-time domestic violence incidents were noted during the COVID-19 pandemic [5].

Adverse psychological outcomes of IPV including depressive symptoms, anxiety symptoms, substance misuse, and even suicidal behaviours are positively associated with IPV victimization [6–8]. Specific violent episodes such as IPV often triggered self-harm, which IPV victims considered as an approach for airing painful emotions caused by abuse or as a last resort to escape by dying when they had no other options and were no longer able to endure IPV [8]. It is worth noting that the impact of IPV victimization extends beyond immediate psychological distress such as different persistent pain on women with a history of IPV victimization [9].

Studies examining gender differences in mental health outcomes of IPV are mixed. For example, one UK-based study demonstrated that physical IPV was significantly linked to psychosis and substance/alcohol disorders in both males and females, yet associations with common mental disorders, posttraumatic stress disorder, and eating disorders were observed exclusively among women; and emotional IPV was related with common mental disorders in both genders [10].

Compared to Western countries, China has a relatively short history of research on IPV. However, the total contribution of IPV research in China has increased significantly during the past two decades [11]. The lifetime prevalence of IPV victimization in the general population in China is reported to range from 10.2% [12] to 65.0% [13], representing a large variation across regions and settings [11]. It shows that around sixty percent of existing articles related to IPV research in China were limited to one gender especially females [11], and only few studies studied the gender differences in the relationships between IPV victimization and different mental health outcomes in the Chinese cultural context. Traditional gender socialization in virtually all cultures stipulates that men have a right to authority in their families and over their female partners [14] without exception in China, which could definitely contribute to the phenomenon of IPV. Domestic violence has previously been viewed more as a private affair within the family rather than a matter of public concern under the traditional Chinese culture [15]. The enduring influence of Confucianism in China continues to shape IPV dynamics [16] especially in the underdeveloped and economically-disadvantaged areas, despite increasing attention being paid to IPV and legal reforms like restraining orders or enactment of laws. Thus, it is of interest to explore the complex interplay between gender, IPV victimization, and different mental health outcomes.

Qinghai is one of the five largest pastoral regions in China with a long history of pastoral livelihoods, while IPV among the local population was barely studied. Therefore, we conducted a cross-sectional study in pastoral region of western China, with the aim to 1) investigate the prevalence of IPV victimization by gender; 2) explore the associations between total, psychological, physical and sexual IPV victimization and mental health outcomes (anxiety symptoms, depressive symptoms, suicide ideation and suicide attempts) and whether these associations differ by gender; and 3) examine the characteristic-specific gender differences in the relationships. Based on a previous study [10], our hypothesis posited that females would report a higher prevalence of IPV victimization compared to males and that IPV victims would be associated with an increased odds of mental health problems and with the effect size differing based on gender.

## Methods

### Study Design and Data Collection

The current study is part of the Survey on Knowledge of Disease Prevention and Control and Quality of Life of residents in pastoralist areas of Qinghai province, which was conducted in Qinghai Province, western China, in September 2021. Qinghai Province is a multiethnic province in China that consisted of 8 prefecture-level divisions (i.e., namely, 45 regional units that are 7 districts, 25 countries, 7 autonomous counties, and 5 county-level cities and 1 administrative zone). We used the stratified random cluster sampling method to recruit participants. First, stratified sampling method was used to select regional units from the 8 prefecture-level divisions, 35 out of 45 were finally and randomly selected. We then performed random sampling method to select the natural villages or communities in each region unit, a cluster sampling method was then used in the selected villages or communities to recruit potential participants.

**FIGURE 1:**
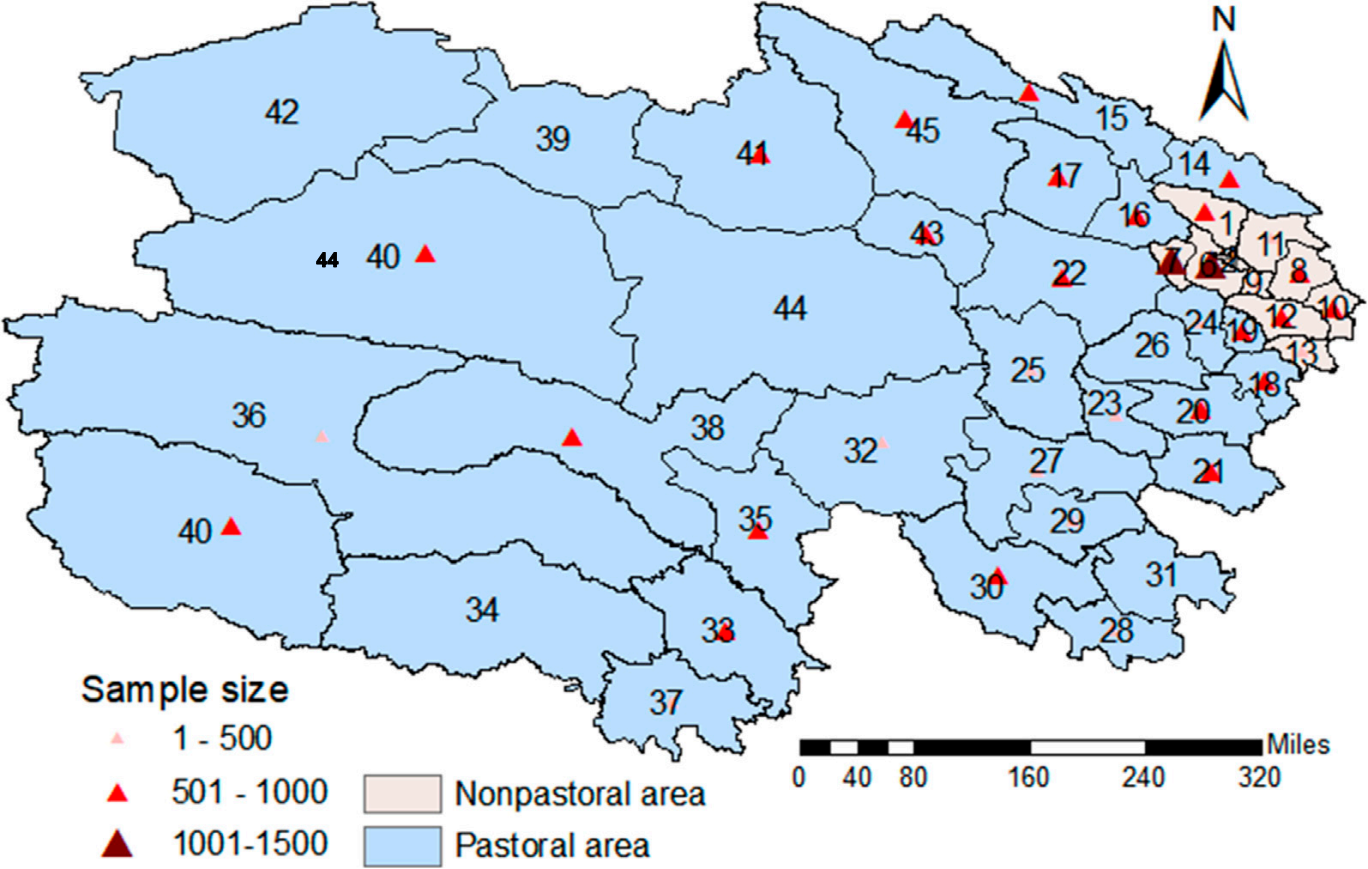
Regional areas involved in this study (Qinghai, China. 2024). Note: 1, Datong Hui and Tu Autonomous County; 2, Chengbei District Xining city; 6, Huangzhong County; 7, Huangyuan County; 8, Ledu District; 9, Ping'an District; 10, Minhe Hui and Tu Autonomous County; 11, Huzhu Tu Autonomous County; 12, Hualong Hui Autonomous County; 13, Xunhua Salar Autonomous County; 14, Menyuan Hui Autonomous County; 15, Qilian County; 16, Haiyan County; 17, Gangca County; 18, Tongren County; 19, Jianzha County; 20, Zeku County; 21, Henan Mongolian Autonomous County; 22, Gonghe County; 23, Tongde County; 24, Guide County; 25, Xinghai County; 26, Guinan County; 27, Maqin County; 28, Banma County; 29, Gande County; 30, Dari County; 31, Jiuzhi County; 32, Maduo County; 33, Yushu City; 34, Zaduo County; 35, Chenduo County; 36, Zhiduo County; 37, Nangqian County; 38, Qumalai County; 39, Da Qaidam Administrative Region Zone.; 40, Ge'ermu City; 41, Delingha City; 42, Mangya City; 43, Wulan County; 44, Dulan County; 45, Tianjun County. 3, Chengxi District of Xining city; 4, Chengdong District of Xining city; 5, Chengzhong District of Xining city, that are next to 2 are not sampled, and were not displayed on the map due to limited spaces, these three areas.

Our trained investigators face to face distributed the self-report questionnaires to potential participants in different scenarios (including household surveys, health education activities and medical checkups, etc.) and collected them after completion; the investigator would help if the participants were not able to literally fill in the questionnaires. Those who met the following inclusion criteria were recruited in this study: 1) aged 18 or above and 2) a permanent resident of Qinghai Province. Figure 1 presents the involved regional areas. We finally received 33,178 paper questionnaires (41,800 were initially planned to send out) yield a response rate of approximately 79.4%. Participants with ≥20% data missing or with extreme values were excluded, yielding 21,824 participants included for analysis.

### Assessment

We collected the following information, basic sociodemographic and individual health status including age years, gender, living areas (non-pastoral/pastoral area), ethnic group, marital status, highest education level, occupation, religious belief, monthly household income [17], significant negative life events (such as parental divorce, major illness of family member, significant property loss, traffic accident or death of a relative) in the last year (no/yes) and chronic medical condition.

#### Exposure

##### Intimate Partner Violence (IPV) Victimization

We evaluated three forms of lifetime IPV victimization, i.e., psychological, physical, and sexual violence [18], by using the following three questions with yes/no options: 1) Have you ever been subjected to psychological violence by your current or ex- partner or spouse (such as insults, yelling, name-calling, denigration, contempt, ridicule, or similar behaviours, etc.)? 2) Have you ever been hit, slapped, kicked, pushed or shoved, or otherwise physically hurt by current or ex- partner or spouse? 3) Has your current or ex- partner or spouse ever forced you to have sex, or has your current or ex- partner or spouse insisted on sex when you did not want to or did not have a condom? Participants with positive answers to at least one type of violence were considered to be “experiencing lifetime IPV victimization.”

#### Mental Health Outcomes

##### Anxiety Symptoms

The validated Chinese version of the 7-item Generalized Anxiety Disorder Scale (GAD-7, Cronbach’s α = 0.88) was used to assess the severity of anxiety symptoms experienced by the participants over the last 2 weeks prior to participation in this survey [19, 20]. It is a self-report screening scale with each item scored from 0 (never) to 3 (very often), and the total score ranges from 0 to 21, with higher total scores indicating more severe anxiety symptoms. We used the cut-off point value of 10 to identify individuals with possible anxiety [19].

##### Depressive Symptoms

The Depression symptoms over the past 2 weeks were evaluated using the validated Chinese version of the Patient Health Questionnaire-9 (PHQ-9, Cronbach’s α = 0.86) [21, 22]. Participants indicated how often they had been bothered by each symptom using a four-point Likert scale ranging from 0 (not at all) to 3 (nearly every day), summing up to an overall score that ranged from 0 to 27. Those who had a score of 10 or greater were considered to have moderate or severe depression symptoms (possible depression) [21].

##### Suicidal Behaviours

We included **suicide ideation** (SI) and **suicide attempts** (SA) as suicidal behaviours in our study, which were assessed by asking the following two yes-no questions, adapted from previous studies [23, 24]:

1) Have you ever seriously thought about committing suicide (SI)?
2) Have you ever attempted suicide (SA)?

### Ethical Considerations

The study protocol was approved by the Ethics Committee of the Medical College of Qinghai University (QHMC201803), and all the participants involved in this survey provided informed consent. We followed the principles of voluntariness and anonymity to perform this study, and the Strengthening the Reporting of Observational Studies in Epidemiology (STROBE) guidelines to report this study [25].

### Statistical Analyses

We compared odds of possible depression, possible anxiety, suicidal ideation and attempts among whole individuals with total IPV with those without IPV, by fitting the multivariate logistic regression models with adjustment for age, gender, living areas, ethnic group, marital status, education level, occupation, religious belief, monthly household income, significant negative life events in the last year and chronic medical condition; and among the female and male genders separately to explore the gender-specific relationships. The results are expressed as odds ratios (ORs) and their 95% confidence intervals (CIs).

In the sensitivity analyses, we used the matched sample generated by performing optimal full matching [26] using the propensity scores followed the propensity score estimation with logistic regression based on the original sample (n = 21,824) to test the robustness of the results from the total IPV. A same set of covariates as added in the aforementioned logistic regressions were used to predict propensity scores of total IPV victimization. Finally, balanced matched sample of 7,029 females and 6,907 males were respectively generated and used in the subsequent sensitivity analyses. The standardized mean differences and visualized plots were used to check on the balance statistics.

We then performed stratification analyses by fitting the models among females and males stratified by each characteristic to examine the characteristic-specific associations between total IPV victimization and each mental health outcome.

The missingness in all models except the sensitivity analyses was handled by executing the multiple imputations by chained equations employing the “mice” R package [27]. Optimal full matching was performed using the “MatchIt” package in R [28], which calls functions from the “optmatch” package [26]. All data were analysed in R version 4.1.2 via RStudio [29], with a significant α threshold of 0.05 (two-tailed).

## Results

### Basic Descriptions

A total of 21,824 participants (female: 44.7%; 40.3 years, SD = 12.6) were included, and 70.2% were living in the pastoral areas, and the minorities other than the Han accounted for 49.6% (n = 10,831). There were 6.7% (95% CI, 6.4%–7.0%) of the participants reported at least one form of lifetime IPV victimization, and the corresponding lifetime prevalence among female and male participants was 7.2% (95% CI, 6.7%–7.7%) and 5.7% (95% CI, 5.3%–6.2%), respectively. Specifically, female experienced higher prevalence of psychological [female: 5.1% (95% CI, 4.7%–5.6%) vs. male: 3.6% (95% CI, 3.3%–4.0%)] and physical [3.7% (95% CI, 3.4%–4.1%) vs. 3.0% (95% CI, 2.6%–3.3%)] IPV victimization than male, but not with higher prevalence of sexual IPV victimization [2.2% (95% CI, 1.9%–2.5%) vs. 2.2% (1.9%–2.4%)]. The basic characteristics and health information of participants are shown in Supplementary Appendix Table 1 and Table 1.

**TABLE 1:** Characteristics of participants with and without intimate partner violence victimization stratified by gender (Qinghai, China. 2024).

	Whole sample^b^ (N = 21,824)	Female participants			Male participants		
		Overall (N = 9,758) n (%)	IPV victimization		Overall (N = 10,116) n (%)	IPV victimization	
			None (N = 9,059) n (%)	Any (N = 699) n (%)		None (N = 9,536) n (%)	Any (N = 580) n (%)
Age							
Mean age (SD)	40.3 (12.6)	38.6 (12.4)	37.4 (10.4)	38.7 (12.5)	41.9 (12.6)	41.9 (12.6)	41.7 (12.5)
<35 years	7,848 (36.0)	4,141 (42.4)	3,829 (42.3)	312 (44.6)	3,062 (30.3)	2,874 (30.1)	188 (32.4)
35–44 years	5,922 (27.1)	2,638 (27.0)	2,430 (26.8)	208 (29.8)	2,832 (28.0)	2,689 (28.2)	143 (24.7)
45–54 years	4,628 (21.2)	1,807 (18.5)	1,672 (18.5)	135 (19.3)	2,458 (24.3)	2,312 (24.2)	146 (25.2)
55 years or more	3,027 (13.9)	1,099 (11.3)	1,057 (11.7)	42 (6.0)	1,668 (16.5)	1,579 (16.6)	89 (15.3)
Missing	399 (1.8)	73 (0.7)	2 (0.3)	71 (0.8)	96 (0.9)	82 (0.9)	14 (2.4)
Living areas							
Non-pastoral area	6,511 (29.8)	3,127 (32.0)	2,910 (32.1)	217 (31.0)	2,953 (29.2)	2,805 (29.4)	148 (25.5)
Pastoral area	15,313 (70.2)	6,631 (68.0)	6,149 (67.9)	482 (69.0)	7,163 (70.8)	6,731 (70.6)	432 (74.5)
Ethnic group							
Han	7,879 (36.1)	3,781 (38.7)	3,560 (39.3)	221 (31.6)	3,616 (35.7)	3,463 (36.3)	153 (26.4)
Others^a^ (Tibetan, Hui, etc.)	10,831 (49.6)	4,880 (50.0)	4,480 (49.5)	400 (57.2)	5,091 (50.3)	4,764 (50.0)	327 (56.4)
Missing	3,114 (14.3)	1,097 (11.2)	1,019 (11.2)	78 (11.2)	1,409 (13.9)	1,309 (13.7)	100 (17.2)
Marital status							
Married	16,921 (77.5)	7,556 (77.4)	7,029 (77.6)	527 (75.4)	7,996 (79.0)	7,581 (79.5)	415 (71.6)
Other	4,450 (20.4)	2,091 (21.4)	1,926 (21.3)	165 (23.6)	1,910 (18.9)	1,759 (18.4)	151 (26.0)
Missing	453 (2.1)	111 (1.1)	104 (1.1)	7 (1.0)	210 (2.1)	196 (2.1)	14 (2.4)
Education level							
No previous education	2,846 (13.0)	1,367 (14.0)	1,257 (13.9)	110 (15.7)	1,209 (12.0)	1,119 (11.7)	90 (15.5)
Primary school	6,427 (29.4)	2,723 (27.9)	2,553 (28.2)	170 (24.3)	3,179 (31.4)	3,006 (31.5)	173 (29.8)
Middle school	6,847 (31.4)	2,764 (28.3)	2,532 (28.0)	232 (33.2)	3,549 (35.1)	3,383 (35.5)	166 (28.6)
College, University or above	4,016 (18.4)	2,308 (23.7)	2,152 (23.8)	156 (22.3)	1,460 (14.4)	1,368 (14.3)	92 (15.9)
Missing	1,688 (7.7)	596 (6.1)	565 (6.2)	31 (4.4)	719 (7.1)	660 (6.9)	59 (10.2)
Occupation							
Unemployed	1,821 (8.3)	876 (9.0)	782 (8.6)	94 (13.4)	750 (7.4)	695 (7.3)	55 (9.5)
Farmer/herder	13,389 (61.3)	5,475 (56.1)	5,141 (56.8)	334 (47.8)	6,711 (66.3)	6,339 (66.5)	372 (64.1)
Civil servants/Personnel in public institutions	2,672 (12.2)	1,504 (15.4)	1,380 (15.2)	124 (17.7)	1,026 (10.1)	963 (10.1)	63 (10.9)
Other	3,508 (16.1)	1,773 (18.2)	1,635 (18.0)	138 (19.7)	1,481 (14.6)	1,401 (14.7)	80 (13.8)
Missing	434 (2.0)	130 (1.3)	121 (1.3)	9 (1.3)	148 (1.5)	138 (1.4)	10 (1.7)
Religious belief							
No	7,910 (36.2)	3,799 (38.9)	3,583 (39.6)	216 (30.9)	3,617 (35.8)	3,465 (36.3)	152 (26.2)
Yes	11,615 (53.2)	5,082 (52.1)	4,648 (51.3)	434 (62.1)	5,516 (54.5)	5,146 (54.0)	370 (63.8)
Missing	2,299 (10.5)	877 (9.0)	828 (9.1)	49 (7.0)	983 (9.7)	925 (9.7)	58 (10.0)
Monthly household income (CNY)							
≤3,000	12,875 (59.0)	5,821 (59.7)	5,393 (59.5)	428 (61.2)	5,862 (57.9)	5,508 (57.8)	354 (61.0)
3,000–5,000	5,316 (24.4)	2,251 (23.1)	2,089 (23.1)	162 (23.2)	2,658 (26.3)	2,529 (26.5)	129 (22.2)
>5,000	3,002 (13.8)	1,434 (14.7)	1,341 (14.8)	93 (13.3)	1,398 (13.8)	1,316 (13.8)	82 (14.1)
Missing	631 (2.9)	252 (2.6)	236 (2.6)	16 (2.3)	198 (2.0)	183 (1.9)	15 (2.6)
Significant negative life events in the last year							
No	19,128 (87.6)	8,725 (89.4)	8,230 (90.8)	495 (70.8)	8,901 (88.0)	8,590 (90.1)	311 (53.6)
Yes	2,213 (10.1)	918 (9.4)	723 (8.0)	195 (27.9)	1,061 (10.5)	810 (8.5)	251 (43.3)
Missing	483 (2.2)	115 (1.2)	106 (1.2)	9 (1.3)	154 (1.5)	136 (1.4)	18 (3.1)
Chronic medical condition							
No	18,581 (85.1)	8,535 (87.5)	7,990 (88.2)	545 (78.0)	8,508 (84.1)	8,128 (85.2)	380 (65.5)
Yes	2,269 (10.4)	874 (9.0)	743 (8.2)	131 (18.7)	1,232 (12.2)	1,076 (11.3)	156 (26.9)
Missing	974 (4.5)	349 (3.6)	326 (3.6)	23 (3.3)	376 (3.7)	332 (3.5)	44 (7.6)

^a^ Other ethnic group includes Tibetan, Hui, etc.

^b^ There were 968 and 1,070 participants with missing data on IPV victimization experience and gender, respectively. Note: CNY, Chinese Yuan; SD, standard deviation.

**FIGURE 2:**
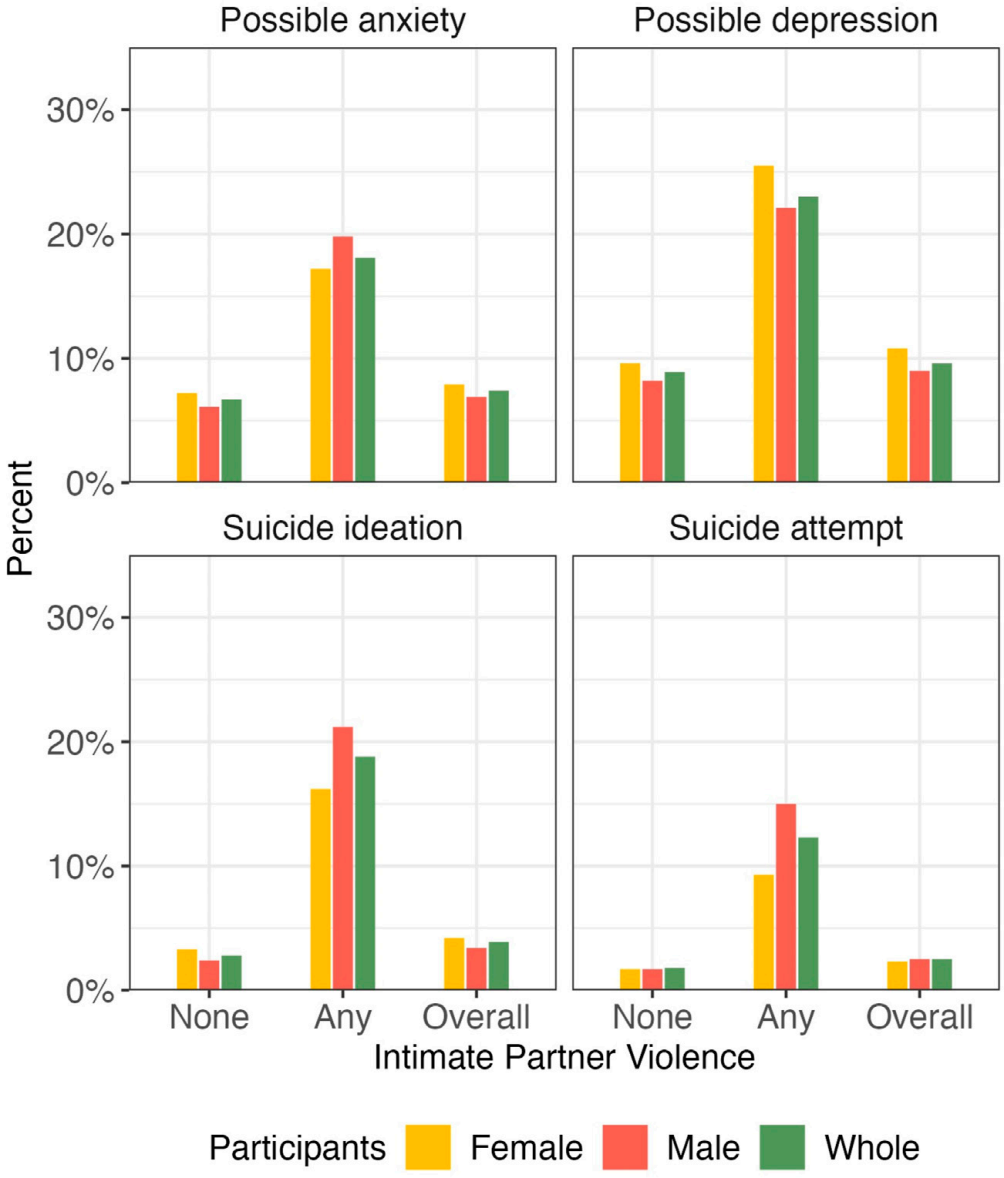
The prevalence of possible anxiety, possible depression, suicide behaviors among participants with and without intimate partner violence victimization among the whole participants and stratified by gender (Qinghai, China. 2024).

Score or prevalence of the four mental health indicators are shown in Supplementary Appendix Tables 2, 3. Compared with IPV-free participants, those from both genders with any form of IPV victimization presented higher prevalence of possible anxiety [female: 17.2% (95% CI, 14.4%–20.2%) vs. 7.2% (95% CI, 6.6%–7.7%); male: 19.8% (95% CI, 16.7%–23.3%) vs. 6.1% (95% CI, 5.7%–6.6%)], possible depression [female: 25.5% (95% CI, 22.3%–28.9%) vs. 9.6% (95% CI, 9.0%–10.3%); male: 22.1% (95% CI, 18.8%–25.7%) vs. 8.2% (95% CI, 7.7%–8.8%)], suicide ideation [female: 16.2% (95% CI, 13.5%–19.1%) vs. 3.3% (95% CI, 2.9%–3.6%; male: 21.2% (95% CI, 17.9%–24.8%) vs. 2.4% (95% CI, 2.1%–2.7%)], and suicide attempts [female: 9.3% (95% CI, 7.3%–11.7%) vs. 1.7% (95% CI, 1.5%–2.0%; male: 15.0% (95% CI, 12.2%–18.2%) vs. 1.7% (95% CI, 1.5%–2.0%)] (Figure 2).

### Gender Differences in the Associations Between IPV Victimization and Mental Health

**FIGURE 3:**
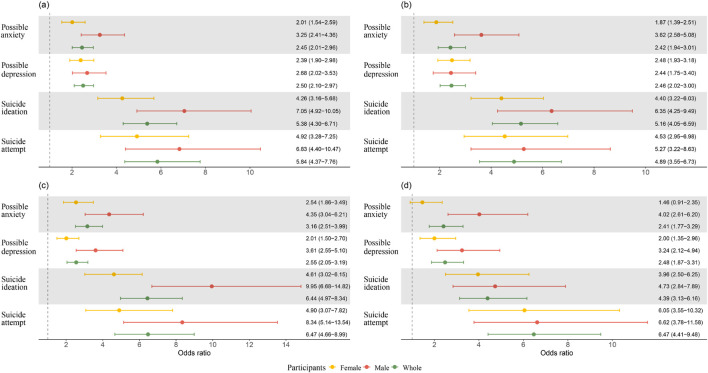
Associations between intimate partner violence victimization and mental health among the whole participants and stratified by gender (Qinghai, China. 2024). Note: Each row represents the result from one model. Multiple imputations were employed. Models among participants stratified by gender were adjusted for age years, living areas, ethnic group, marital status, education level, occupation, religion belief, monthly household income, significant negative life events in the last year and chronic medical condition. Genders was added and adjusted for models among whole participants. **(A)** Total IPV. **(B)** Psychological IPV. **(C)** Physical IPV. **(D)** Sexual IPV.

IPV victimization was positively associated with the four mental health indicators among both female and male participants. Supplementary Appendix Tables 4–6 show the results of the full models for total IPV, the ORs among males was greater than that among females, while the confidence intervals for estimates overlapped. Specifically, male participants with psychological (male: OR = 3.62, 95% CI: 2.58–5.08 vs. female: OR = 1.87, 95% CI: 1.39–2.51) or sexual (OR = 4.02, 95% CI: 2.61–6.20 vs. OR = 1.46, 95% CI: 0.91–2.35) IPV victimization presented greater odds of possible anxiety than females; males with physical IPV victimization showed greater likelihood of with suicide ideation than females (OR = 9.95, 95% CI: 6.68–14.82 vs. OR = 4.61, 95% CI: 3.02–6.15). Figures 3A–D shows the gender-specific associations between total, psychological, physical and sexual IPV victimization and the four mental health indicators.

We conducted the sensitivity analysis for the total IPV experiences among 7,029 female and 6,907 male participants that were respectively yielded by applying optimal full matching analysis, the standardized mean differences were all less than 0.1 indicating good balance statistics (Supplementary Appendix Figures 1, 2). The descriptions of participants with and without IPV victimization among both gender after matching are shown in Supplementary Appendix Table 7. Based on the matched dataset, we found similar patterns on the gender differences in the associations between total IPV victimization and mental health indicators in comparison with models fitted with the original dataset. Please see Supplementary Appendix Tables 8–10 for details.

### Characteristic-Specific Gender Differences

**FIGURE 4:**
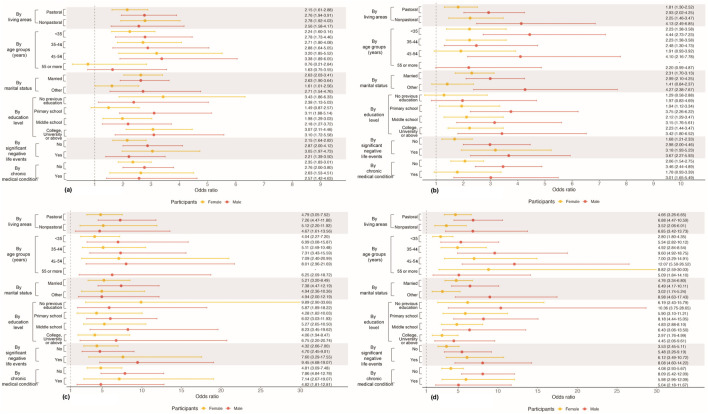
Associations between intimate partner violence victimization and mental health among the whole participants among female and male participants stratified by each characteristic (Qinghai, China. 2024). Note: Each row represents the result from one model. Multiple imputations were employed. Models among female and male participants stratified by each characteristic were adjusted for covariates apart from the characteristic and gender. **(A)** Possible anxiety. **(B)** Possible depression. **(C)** Suicide ideation. **(D)** Suicide attempt.

There was no gender difference with statistical significance for characteristic-specific associations between total IPV victimization and the four mental health outcomes. Figures 4A–D shows the associations stratified by part of the characteristics including living areas, age groups, marital status, education level, significant negative life events and chronic medical condition (see Supplementary Appendix Tables 11–14 for results of all characteristics). All the characteristic-specific associations between IPV victimization and suicide ideation (Figure 4C) and suicide attempt (Figure 4D) were statistically significant. The associations with possible anxiety were not statistically significant in individuals aged 55 years or more among both gender, and female individuals with primary school educational achievement. There was no significance in the association with possible depression in individuals without previous education of both gender, and in female participants aged 45–54 years, with other marital status and with chronic medical condition.

## Discussion

Based on this large-scale population-based study, we found that females had a higher lifetime prevalence of being a victim of total IPV than males in China; specifically, female experienced higher prevalence of psychological and physical IPV victimization than male, but no significant gender difference in prevalence of sexual IPV victimization. Total IPV victimization was positively associated with reporting possible anxiety and depression, suicide ideation and suicide attempt in both females and males, which was reconfirmed in the optimal full matching-based data. Male participants with psychological or sexual IPV victimization presented greater odds of possible anxiety than females; males with physical IPV victimization showed greater likelihood of with suicide ideation than females. We found no difference with statistical significance for gender-specific associations between total IPV victimization and the four mental health outcomes. Our findings help to better understand the characteristic-specific gender differences in the prevalence of IPV victimization and its relationships to mental health problems in a less studied region, and provide evidence on how to tailor IPV-related preventive strategies and multidisciplinary interventions to the vulnerable group at risk.

The prevalence of total IPV victimization was 6.7% in our sample, which was lower than the general lifetime prevalence of 10.2%–65.0% reported in a review of Chinese population studies [11], and lower than a global prevalence of 24% of women aged 15–19 years [2]. Compared with data since COVID-19 lockdown began especially at the early stage, our result was lower than 11.3% of participants reporting psychological or physical intimate partner abuse on at least one occasion in the UK [30], 18% of individuals with IPV victimization in the United States [31]. Social isolation and substantial self-quarantine at home led to constant contact between victims and perpetrators, IPV victims are unable to safely connect with services (such as domestic-violence hotlines), which could result in increased violent events and decreased reports [3, 4]. Besides, the role of stigma and stigmatization could limit individuals’ willingness to disclose their true experiences, especially in the underdeveloped and economically-disadvantaged areas and our investigators collected the written questionnaires face-to-face.

Our findings echo the majority of studies showing that females have a higher prevalence of IPV victimization than males. As a review showed, IPV in mainland China is not a unitary phenomenon, the prevalence of different types of IPV reflects gender differences in both victimization and perpetration [32], by which our findings could help to extend the existing literature. Previous studies have revealed the mixed findings about IPV prevalence across gender [33], disparities in the samples (i.e., community- or agency- based) and differences in the likelihood of reporting and the patterns of IPV reporting across gender could partially explain the controversy over gender asymmetry [33]. Inconsistent measurements to assess IPV and diverse cultural contexts could also play a role in the gender difference of IPV prevalence. IPV-victimized women experienced feelings of shame, stigma, and “losing face” and had little social support [34], which could possibly lead to underreporting bias, although reporting biases are primarily inevitable [33].

Being a IPV victim was positively associated with anxiety and depressive symptoms, suicide ideation and suicide attempt, which coincide with the broader findings not only in the general population but also in the maternity population [6, 7, 35–38]. IPV can diminish individuals’ self-esteem and sense of identity [39], which could result in feelings of worthlessness and hopelessness. The consistently strong relationship between intimate partner abuse and suicidality held irrespective of sample source, study design and assessment methods [37]. It showed that 50% of those experienced psychological or physical IPV during COVID-19 lockdown had suicidal or self-harm thoughts and 25% of them had self-harm behaviors [40]. While a previous systematic review indicated the clear evidence of IPV’s association with suicide attempts in females but not in males [41].

Although we found that females reported a higher prevalence of total, psychological and physical IPV victimization than males, male participants with psychological or sexual IPV victimization presented greater odds of possible anxiety than females and with physical IPV victimization showed greater likelihood of with suicide ideation than females. With limited available comparisons, one study in Spain indicated no difference with statistical significance for gender-specific associations between total IPV and suicide ideation (OR = 6.3, 95% CI: 4.5–8.9 vs. OR = 5.5, 95% CI: 3.5–8.6), partially consistent with our results, the 95% CIs of both gender were also overlapped; but they identified greater associations with posttraumatic stress and depressive disorders in men than women [42], since we found no significant gender difference with possible depression. From a biological point of view, the hypothalamic-pituitary-adrenal (HPA) axis in females may be more susceptible to stress-induced dysregulation than in males [43]. But females express more emotion than males [44], and may appraise abuse differently than males [45], which could partly help to explain the finding. Sociologically, individuals may employ different coping mechanisms as responses based on their gender roles and societal expectations. For example, due to societal expectations of masculinity [46], the particularly significant impact of self-worth for males [46], and the stigma surrounding male victimization [47], males may be less likely to seek social support or professional help and escape the abusive relationship, which could lead to a greater accumulation of poor mental health over time. In addition, there were disparities regarding the explanations of IPV based on the feminist and patriarchal theory [14]. The above-discussed context helped to explain the differences of IPV victimization and its relationships with mental health in males and females.

IPV’s side effects were not limited to the poor psychological outcomes, but decreases in relationship satisfaction. Therefore, possible prevention programs to reduce the likelihood of and the detrimental effects of IPV should be tailored to respond to the specific patterns of violence in various contexts [48]. Although the term “IPV” was barely studied until 2004 in China, IPV is not a new phenomenon, and there has been an increase in public awareness and a growing political consensus regarding necessary action to address IPV over the past few decades [11]. For instance, China’s first law against domestic violence was issued on 27 December 2015, which indicated the definition of domestic violence and specific guidance in terms of the prevention and implementation of strategies that can reduce domestic violence (National Law against Domestic Violence of the People’s Republic of China, 2015). Efforts have been made at multiple levels, including policy and academic aspects, etc., to reduce the incidence and negative impacts of IPV in China, but more attention is needed, especially based on gender-specific features.

It has been noted that fewer quantitative studies of IPV in China are currently conducted in rural areas [11], our findings with a large sample ideally extended the literature given that 70.2% of the study sample was from pastoral areas and with 10,166 male individuals. In addition, our study provided evidence in terms of the significant gender differences in the prevalence of different subtypes of IPV victimization and their specific associations with possible anxiety and suicide ideation. However, several limitations that should be noted. First, although we took advantage of the optimal full matching method to reduce bias, it is impossible to capture causality due to the cross-sectional nature of the study. Second, although we assessed three aspects of IPV separately, only simple questions rather than widely used scales were applied, which could lower the efficacy and make it impossible to make direct comparisons with other existing studies using structured scales, and will be considered in our further longitudinal and intervention studies. Third, follow-ups were not designed, especially for those with negative exposures, while our study investigators have reminded the participants that if they seek for it, services and support will be definitely provided by the local professionals in the department of public health and other social service agencies.

In conclusion, our study revealed higher prevalence of total, psychological and physical IPV victimization experienced in females than males in China, but no difference in sexual IPV victimization. There were positive associations between IPV victimization and anxiety symptoms, depressive symptoms, suicide ideation and suicide attempts; gender specifically, male participants with psychological or sexual IPV victimization presented greater odds of possible anxiety than females; males with physical IPV victimization showed greater likelihood of with suicide ideation than females. Programs and policies should be tailored to prevent and reduce the likelihood of and the detrimental effects of IPV victimization in various contexts.

## Data Availability

More information can be obtained by email to liser@outlook.com/li.lu@xjtu.edu.cn.
