# Supplementary material for: Gender Differences in Intimate Partner Violence Victimization and Its Relationships With Anxiety, Depression Symptoms and Suicide Behaviours in China
Source: Int J Public Health. 2025 Feb 18;70:1607953. doi: 10.3389/ijph.2025.1607953 (PMC11876969; doi:10.3389/ijph.2025.1607953)
Supplement: Supplementary file 1 [file DataSheet1.doc]

Appendix figure 1: Standardized mean difference after optimal full matching between samples among female participants (Qinghai, China, 2024)

Appendix figure 2: Standardized mean difference after optimal full matching between samples among male participants (Qinghai, China, 2024)

Appendix table 1: Basic characteristics of the whole participants with and without victimization (Qinghai, China, 2024)

Appendix table 2: Mental health among participants with and without intimate partner violence victimization (Qinghai, China, 2024)

Appendix table 3: Mental health among participants with and without intimate partner violence victimization stratified by gender (Qinghai, China, 2024)

Appendix table 4: The association between intimate partner violence victimization and mental health among the whole population (Qinghai, China. 2024)

Appendix table 5: The association between intimate partner violence victimization and mental health among the female population (Qinghai, China. 2024)

Appendix table 6: The association between intimate partner violence victimization and mental health among the **male** population (Qinghai, China. 2024)

Appendix Table 7: Comparison of characteristics among female and male participants between those with and without intimate partner violence victimization after optimal full matching (Qinghai, China. 2024)

Appendix table 8: Associations between intimate partner violence victimization and mental health problems using propensity score matching among the whole participants (N=13969) (Qinghai, China. 2024)

Appendix table 9: Associations between intimate partner violence victimization and mental health problems using propensity score matching among the female participants (N=7029) (Qinghai, China. 2024)

Appendix table 10: Associations between intimate partner violence victimization and mental health problems using propensity score matching among the male participants (N=6907) (Qinghai, China. 2024)

Appendix table 11: Gender-stratified associations between intimate partner violence victimization and possible anxiety by each characteristic (Qinghai, China. 2024)

Appendix table 12: Gender-stratified associations between intimate partner violence victimization and possible depression by each characteristic (Qinghai, China. 2024)

Appendix table 13: Gender-stratified associations between intimate partner violence victimization and suicide ideation by each characteristic (Qinghai, China. 2024)

Appendix table 14: Gender-stratified associations between intimate partner violence victimization and suicide attempt by each characteristic (Qinghai, China. 2024)


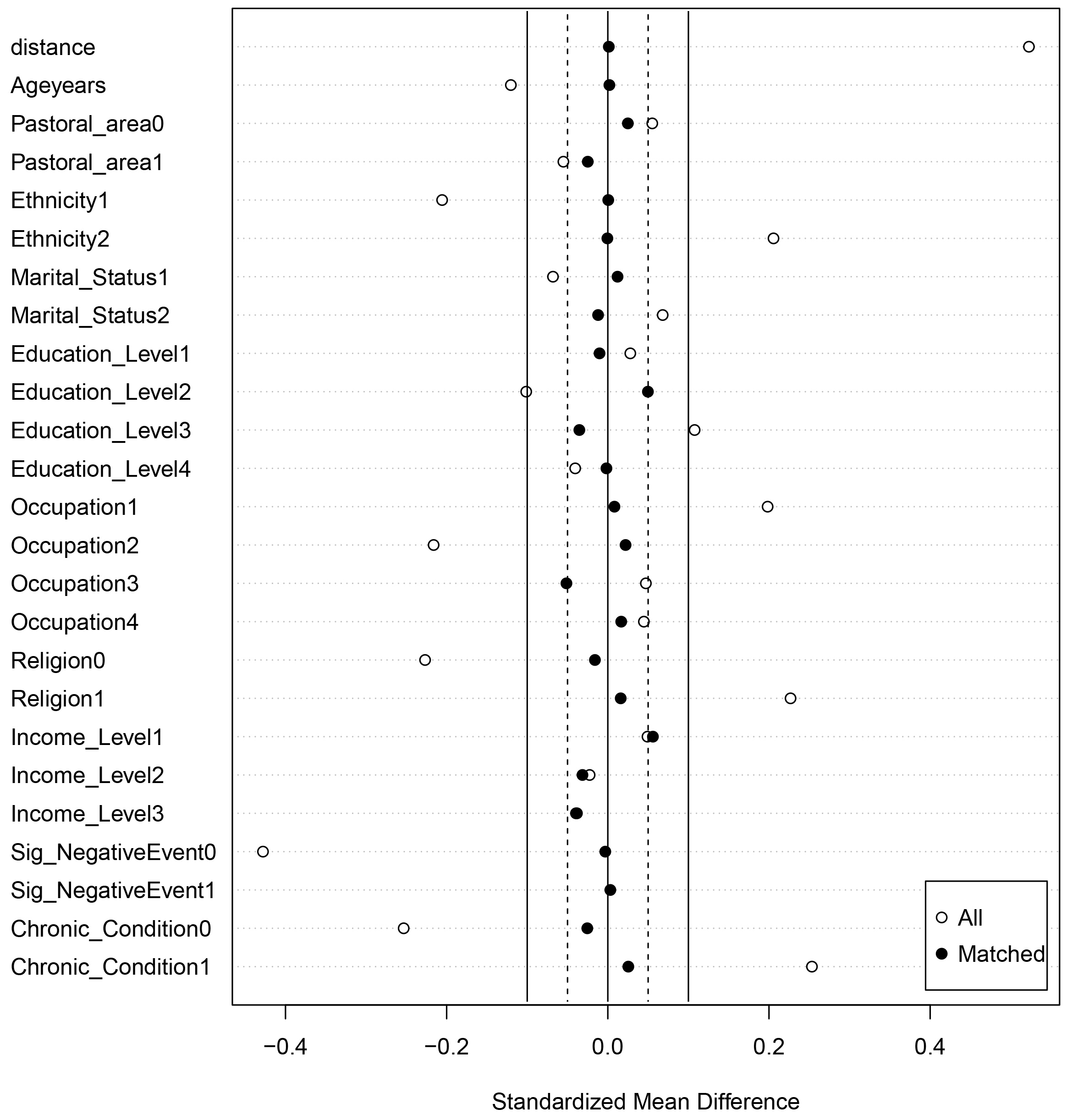


Appendix figure 1: Standardized mean difference after optimal full matching between samples among **female** participants (Qinghai, China. 2024)


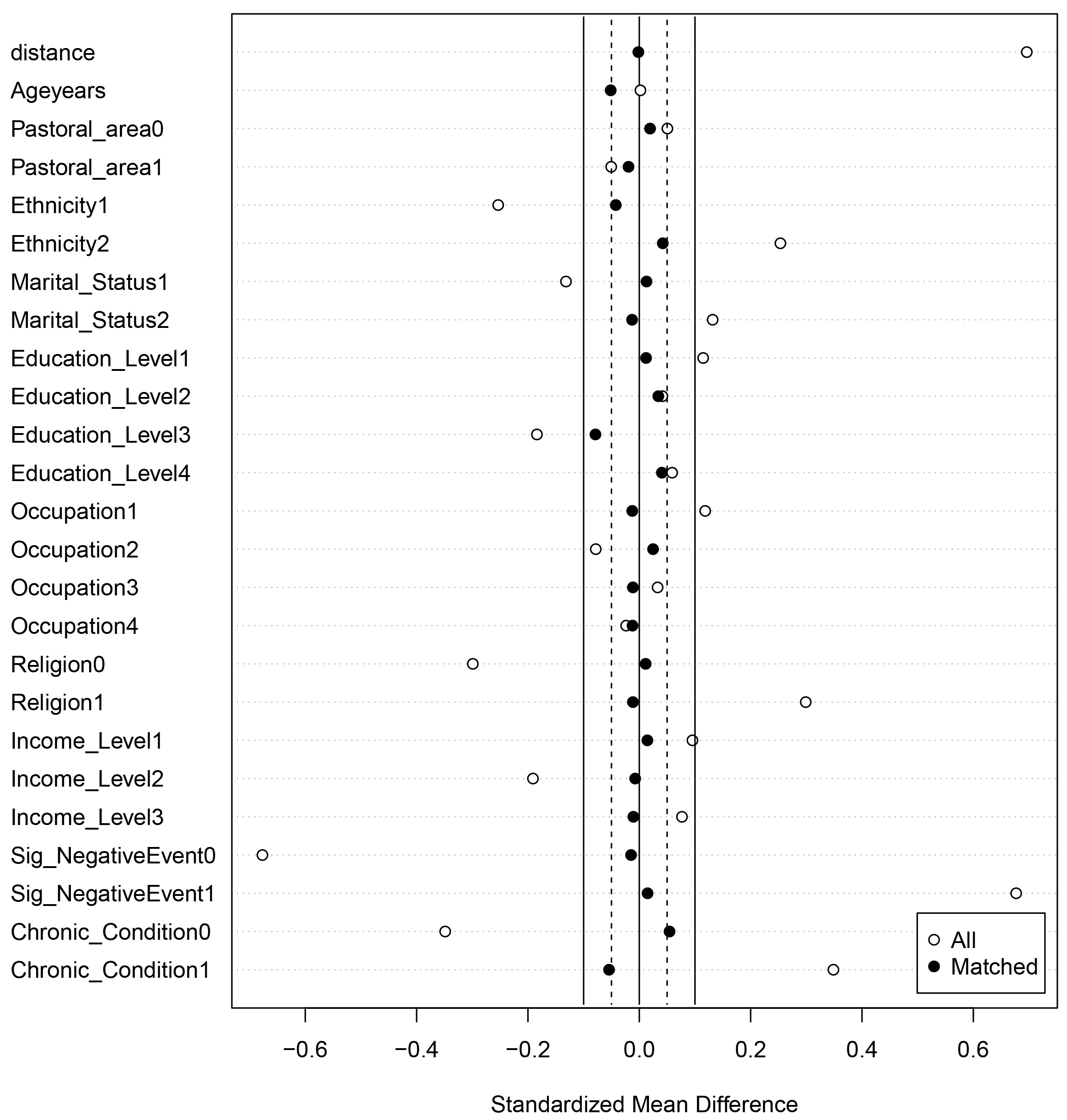


Appendix figure 2: Standardized mean difference after optimal full matching between samples among **male** participants (Qinghai, China. 2024)

Appendix table 1: Basic characteristics of the whole participants with and without victimization (Qinghai, China. 2024)

|  | **Overall (N=21824)** | **Intimate Partner Violence** | |
| --- | --- | --- | --- |
|  |  | **None (N=19452)** | **Any (N=1394)** |
| **Age years** |  |  |  |
| Mean (SD) | 40.3 (12.6) | 40.3 (12.7) | 39.3 (11.6) |
| Missing | 399 (1.8%) | 284 (1.5%) | 67 (4.8%) |
| **Gender** |  |  |  |
| Female | 10163 (46.6%) | 9059 (46.6%) | 699 (50.1%) |
| Male | 10591 (48.5%) | 9536 (49.0%) | 580 (41.6%) |
| Missing | 1070 (4.9%) | 857 (4.4%) | 115 (8.2%) |
| **Living areas** |  |  |  |
| Nonpastoral area | 6511 (29.8%) | 5912 (30.4%) | 383 (27.5%) |
| Pastoral area | 15313 (70.2%) | 13540 (69.6%) | 1011 (72.5%) |
| **Ethnic group** |  |  |  |
| Han | 7879 (36.1%) | 7252 (37.3%) | 395 (28.3%) |
| Others ^ (Tibetan, Hui, etc.) | 10831 (49.6%) | 9613 (49.4%) | 801 (57.5%) |
| Missing | 3114 (14.3%) | 2587 (13.3%) | 198 (14.2%) |
| **Marital status** |  |  |  |
| Married | 16921 (77.5%) | 15250 (78.4%) | 987 (70.8%) |
| Other | 4450 (20.4%) | 3853 (19.8%) | 380 (27.3%) |
| Missing | 453 (2.1%) | 349 (1.8%) | 27 (1.9%) |
| **Education level** |  |  |  |
| No previous education | 2846 (13.0%) | 2524 (13.0%) | 208 (14.9%) |
| Primary school | 6427 (29.4%) | 5738 (29.5%) | 372 (26.7%) |
| Middle school | 6847 (31.4%) | 6131 (31.5%) | 451 (32.4%) |
| College, University or above | 4016 (18.4%) | 3658 (18.8%) | 263 (18.9%) |
| Missing | 1688 (7.7%) | 1401 (7.2%) | 100 (7.2%) |
| **Occupation** |  |  |  |
| Unemployed | 1821 (8.3%) | 1554 (8.0%) | 165 (11.8%) |
| Farmer/herder | 13389 (61.3%) | 12009 (61.7%) | 756 (54.2%) |
| Civil servants/Personnel in public institutions | 2672 (12.2%) | 2421 (12.4%) | 197 (14.1%) |
| Other | 3508 (16.1%) | 3166 (16.3%) | 224 (16.1%) |
| Missing | 434 (2.0%) | 302 (1.6%) | 52 (3.7%) |
| **Religious belief** |  |  |  |
| No | 7910 (36.2%) | 7285 (37.5%) | 388 (27.8%) |
| Yes | 11615 (53.2%) | 10289 (52.9%) | 886 (63.6%) |
| Missing | 2299 (10.5%) | 1878 (9.7%) | 120 (8.6%) |
| **Monthly household income (CNY)** |  |  |  |
| ≤ 3000 | 12875 (59.0%) | 11433 (58.8%) | 859 (61.6%) |
| 3000 - 5000 | 5316 (24.4%) | 4796 (24.7%) | 311 (22.3%) |
| > 5000 | 3002 (13.8%) | 2755 (14.2%) | 186 (13.3%) |
| Missing | 631 (2.9%) | 468 (2.4%) | 38 (2.7%) |
| **Significant negative life events in the last year** |  |  |  |
| No | 19128 (87.6%) | 17589 (90.4%) | 876 (62.8%) |
| Yes | 2213 (10.1%) | 1601 (8.2%) | 484 (34.7%) |
| Missing | 483 (2.2%) | 262 (1.3%) | 34 (2.4%) |
| **Chronic medical condition** |  |  |  |
| No | 18581 (85.1%) | 16868 (86.7%) | 1016 (72.9%) |
| Yes | 2269 (10.4%) | 1894 (9.7%) | 306 (22.0%) |
| Missing | 974 (4.5%) | 690 (3.5%) | 72 (5.2%) |

^: Other ethnic group includes Tibetan, Hui, etc.

Note: CNY, Chinese Yuan; SD, Standard deviation.

Appendix table 2: Mental health among participants with and without intimate partner violence victimization (Qinghai, China. 2024)

|  | **Overall (N=21824)** | **Intimate Partner Violence** | |
| --- | --- | --- | --- |
|  |  | **None (N=19452)** | **Any (N=1394)** |
| **GAD-7 score** |  |  |  |
| Mean (SD) | 2.46 (3.98) | 2.33 (3.84) | 4.63 (5.26) |
| Missing | 1139 (5.2) | 895 (4.6) | 112 (8.0) |
| **Possible anxiety** (GAD-7 score ≥10) |  |  |  |
| No | 19072 (87.4) | 17261 (88.7) | 1029 (73.8) |
| Yes | 1613 (7.4) | 1296 (6.7) | 253 (18.1) |
| Missing | 1139 (5.2) | 895 (4.6) | 112 (8.0) |
| **PHQ-9 score** |  |  |  |
| Mean (SD) | 3.14 (4.78) | 3.02 (4.63) | 5.61 (6.37) |
| Missing | 1140 (5.2) | 935 (4.8) | 102 (7.3) |
| **Possible depression** (PHQ-9 score ≥10) |  |  |  |
| No | 18587 (85.2) | 16791 (86.3) | 972 (69.7) |
| Yes | 2097 (9.6) | 1726 (8.9) | 320 (23.0) |
| Missing | 1140 (5.2) | 935 (4.8) | 102 (7.3) |
| **Suicide ideation** |  |  |  |
| No | 20979 (96.1) | 18900 (97.2) | 1132 (81.2) |
| Yes | 845 (3.9) | 552 (2.8) | 262 (18.8) |
| **Suicide attempts** |  |  |  |
| No | 19948 (91.4) | 18002 (92.5) | 1103 (79.1) |
| Yes | 542 (2.5) | 341 (1.8) | 172 (12.3) |
| Missing | 1334 (6.1) | 1109 (5.7) | 119 (8.5) |

GAD-7, Generalized Anxiety Disorder Scale-7; PHQ-9, Patient Health Questionnaire-9.

SD: Standard deviation.

Appendix table 3: Mental health among participants with and without intimate partner violence victimization stratified by gender (Qinghai, China. 2024)

|  | **Female** participants | | |  | **Male** participants | | |
| --- | --- | --- | --- | --- | --- | --- | --- |
|  | Overall (N=9758)  n (%) | IPV victimization | |  | Overall (N=10116)  n (%) | IPV victimization | |
|  |  | None  (N=9059)  n (%) | Any (N=699)  n (%) |  |  | None (N=9536)  n (%) | Any (N=580)  n (%) |
| **GAD-7 score** |  |  |  |  |  |  |  |
| Mean (SD) | 2.68 (4.09) | 2.53 (3.97) | 4.72 (5.07) |  | 2.26 (3.82) | 2.12 (3.66) | 4.62 (5.43) |
| Missing | 384 (3.9) | 336 (3.7) | 48 (6.9) |  | 457 (4.5) | 412 (4.3) | 45 (7.8) |
| **Possible anxiety** (GAD-7 score ≥10) |  |  |  |  |  |  |  |
| No | 8606 (88.2) | 8075 (89.1) | 531 (76.0) |  | 8960 (88.6) | 8540 (89.6) | 420 (72.4) |
| Yes | 768 (7.9) | 648 (7.2) | 120 (17.2) |  | 699 (6.9) | 584 (6.1) | 115 (19.8) |
| Missing | 384 (3.9) | 336 (3.7) | 48 (6.9) |  | 457 (4.5) | 412 (4.3) | 45 (7.8) |
| **PHQ-9 score** |  |  |  |  |  |  |  |
| Mean (SD) | 3.46 (4.90) | 3.26 (4.73) | 5.99 (6.30) |  | 2.97 (4.69) | 2.82 (4.52) | 5.57 (6.53) |
| Missing | 459 (4.7) | 418 (4.6) | 41 (5.9) |  | 502 (5.0) | 453 (4.8) | 49 (8.4) |
| **Possible depression** (PHQ-9 score ≥10) |  |  |  |  |  |  |  |
| No | 8248 (84.5) | 7768 (85.7) | 480 (68.7) |  | 8703 (86.0) | 8300 (87.0) | 403 (69.5) |
| Yes | 1051 (10.8) | 873 (9.6) | 178 (25.5) |  | 911 (9.0) | 783 (8.2) | 128 (22.1) |
| Missing | 459 (4.7) | 418 (4.6) | 41 (5.9) |  | 502 (5.0) | 453 (4.8) | 49 (8.4) |
| **Suicide ideation** |  |  |  |  |  |  |  |
| No | 9350 (95.8) | 8764 (96.7) | 586 (83.8) |  | 9768 (96.6) | 9311 (97.6) | 457 (78.8) |
| Yes | 408 (4.2) | 295 (3.3) | 113 (16.2) |  | 348 (3.4) | 225 (2.4) | 123 (21.2) |
| **Suicide attempts** |  |  |  |  |  |  |  |
| No | 9039 (92.6) | 8459 (93.4) | 580 (83.0) |  | 9215 (91.1) | 8781 (92.1) | 434 (74.8) |
| Yes | 222 (2.3) | 157 (1.7) | 65 (9.3) |  | 250 (2.5) | 163 (1.7) | 87 (15.0) |
| Missing | 497 (5.1) | 443 (4.9) | 54 (7.7) |  | 651 (6.4) | 592 (6.2) | 59 (10.2) |

GAD-7, Generalized Anxiety Disorder Scale-7; PHQ-9, Patient Health Questionnaire-9. SD: Standard deviation.

Appendix table 4: The association between intimate partner violence victimization and mental health among the **whole** population (Qinghai, China. 2024)

| **Characteristics** | **Anxiety symptoms** | | |  | **Depression symptoms** | | |  | **Suicide ideation** | | |  | **Suicide attempts** | | |
| --- | --- | --- | --- | --- | --- | --- | --- | --- | --- | --- | --- | --- | --- | --- | --- |
|  | **Overall  (N=20685)**  **[n, (%)]** | **GAD-7 score ≥10** | |  | **Overall (N=20684)**  **[n, (%)]** | **PHQ-9 score ≥10** | |  | **Overall (N=21824)**  **[n, (%)]** | **Suicide ideation (Yes)** | |  | **Overall (N=20490)**  **[n, (%)]** | **Suicide attempts (Yes)** | |
|  |  | **(n=1613, 7.8)**  **[n, (%)]** | **OR (95% CI) ^#^** |  |  | **(n=2097, 10.1)**  **[n, (%)]** | **OR (95% CI) ^#^** |  |  | **(n=845, 3.9)**  **[n, (%)]** | **OR (95% CI) ^#^** |  |  | **(n=542, 2.6)**  **[n, (%)]** | **OR (95% CI) ^#^** |
| **IPV victimization** |  |  |  |  |  |  |  |  |  |  |  |  |  |  |  |
| No | 18557 (89.7) | 1296 (80.3) | Ref |  | 18517 (89.5) | 1726 (82.3) | Ref |  | 19452 (89.1) | 552 (65.3) | Ref |  | 18343 (89.5) | 341 (62.9) | Ref |
| Yes | 1282 (6.2) | 253 (15.7) | **2.45 (2.02-2.97) ^***^** |  | 1292 (6.2) | 320 (15.3) | **2.50 (2.10-2.98) ^***^** |  | 1394 (6.4) | 262 (31.0) | **5.38 (4.30-6.73) ^***^** |  | 1275 (6.2) | 172 (31.7) | **5.84 (4.38-7.79) ^***^** |
| **Age years** (Mean, SD) | 40.3 (12.7) | 39.2 (13.1) | 0.99 (0.99-1.001) |  | 40.3 (12.7) | 39.0 (12.9) | 0.99 (0.99-1.003) |  | 40.3 (12.6) | 38.1 (12.1) | 0.99 (0.98-0.998) ^*^ |  | 40.3 (12.7) | 37.9 (12.0) | 0.99 (0.98-0.999) ^*^ |
| **Gender** |  |  |  |  |  |  |  |  |  |  |  |  |  |  |  |
| Male | 10075 (48.7) | 745 (46.2) | Ref |  | 10030 (48.5) | 939 (44.8) | Ref |  | 10591 (48.5) | 363 (43.0) | Ref |  | 9881 (48.2) | 260 (48.0) | Ref |
| Female | 9725 (47.0) | 782 (48.5) | 1.10 (0.97-1.25) |  | 9668 (46.7) | 1073 (51.2) | 1.11 (0.99-1.24) |  | 10163 (46.6) | 420 (49.7) | 1.21 (0.99-1.46) |  | 9633 (47.0) | 234 (43.2) | 0.88 (0.69-1.12) |
| **Living areas** |  |  |  |  |  |  |  |  |  |  |  |  |  |  |  |
| Nonpastoral area | 6297 (30.4) | 502 (31.1) | Ref |  | 6234 (30.1) | 736 (35.1) | Ref |  | 6511 (29.8) | 184 (21.8) | Ref |  | 6266 (30.6) | 73 (13.5) | Ref |
| Pastoral area | 14388 (69.6) | 1111 (68.9) | 1.07 (0.92-1.24) |  | 14450 (69.9) | 1361 (64.9) | 1.07 (0.92-1.24) |  | 15313 (70.2) | 661 (78.2) | 1.20 (0.96-1.50) |  | 14224 (69.4) | 469 (86.5) | 2.47 (1.78-3.43) ^***^ |
| **Ethnic group** |  |  |  |  |  |  |  |  |  |  |  |  |  |  |  |
| Han | 7541 (36.5) | 633 (39.2) | Ref |  | 7542 (36.5) | 801 (38.2) | Ref |  | 7879 (36.1) | 270 (32.0) | Ref |  | 7474 (36.5) | 153 (28.2) | Ref |
| Others ^ | 10282 (49.7) | 757 (46.9) | 0.78 (0.63-0.98) ^*^ |  | 10230 (49.5) | 1044 (49.8) | 0.95 (0.79-1.16) |  | 10831 (49.6) | 428 (50.7) | 1.03 (0.74-1.43) |  | 10222 (49.9) | 277 (51.1) | 0.70 (0.45-1.08) |
| **Marital status** |  |  |  |  |  |  |  |  |  |  |  |  |  |  |  |
| Married | 16045 (77.6) | 1109 (68.8) | Ref |  | 16092 (77.8) | 1490 (71.1) | Ref |  | 16921 (77.5) | 549 (65.0) | Ref |  | 15978 (78.0) | 365 (67.3) | Ref |
| Other | 4241 (20.5) | 466 (28.9) | 1.39 (1.20-1.62) ^***^ |  | 4177 (20.2) | 572 (27.3) | 1.24 (1.08-1.42) ^**^ |  | 4450 (20.4) | 281 (33.3) | 1.46 (1.18-1.81) ^***^ |  | 4093 (20.0) | 163 (30.1) | 1.09 (0.81-1.45) |
| **Education level** |  |  |  |  |  |  |  |  |  |  |  |  |  |  |  |
| No previous education | 2715 (13.1) | 189 (11.7) | Ref |  | 2726 (13.2) | 276 (13.2) | Ref |  | 2846 (13.0) | 95 (11.2) | Ref |  | 2673 (13.0) | 68 (12.5) | Ref |
| Primary school | 6118 (29.6) | 511 (31.7) | 1.25 (1.01-1.55) ^*^ |  | 5989 (29.0) | 572 (27.3) | 1.08 (0.89-1.31) |  | 6427 (29.4) | 244 (28.9) | 1.32 (0.95-1.84) |  | 6040 (29.5) | 186 (34.3) | 1.50 (1.01-2.23) ^*^ |
| Middle school | 6485 (31.4) | 427 (26.5) | 0.76 (0.59-0.96) ^*^ |  | 6497 (31.4) | 569 (27.1) | 0.81 (0.66-0.996) ^*^ |  | 6847 (31.4) | 205 (24.3) | 0.93 (0.65-1.33) |  | 6436 (31.4) | 129 (23.8) | 0.92 (0.59-1.44) |
| College, University or above | 3867 (18.7) | 402 (24.9) | 1.12 (0.83-1.50) |  | 3881 (18.8) | 577 (27.5) | 1.09 (0.84-1.41) |  | 4016 (18.4) | 224 (26.5) | 1.35 (0.87-2.09) |  | 3818 (18.6) | 108 (19.9) | 1.34 (0.76-2.36) |
| **Occupation** |  |  |  |  |  |  |  |  |  |  |  |  |  |  |  |
| Unemployed | 1700 (8.2) | 165 (10.2) | Ref |  | 1662 (8.0) | 237 (11.3) | Ref |  | 1821 (8.3) | 87 (10.3) | Ref |  | 1689 (8.2) | 62 (11.4) | Ref |
| Farmer/herder | 12722 (61.5) | 848 (52.6) | 0.75 (0.59-0.94) ^*^ |  | 12735 (61.6) | 1007 (48.0) | 0.61 (0.50-0.75) ^***^ |  | 13389 (61.3) | 433 (51.2) | 0.97 (0.68-1.39) |  | 12572 (61.4) | 319 (58.9) | 1.03 (0.67-1.57) |
| Civil servants/Personnel in Public institutions | 2573 (12.4) | 253 (15.7) | 1.20 (0.87-1.64) |  | 2584 (12.5) | 386 (18.4) | 1.21 (0.92-1.59) |  | 2672 (12.2) | 143 (16.9) | 1.35 (0.85-2.15) |  | 2496 (12.2) | 61 (11.3) | 0.84 (0.45-1.57) |
| Other | 3318 (16.0) | 312 (19.3) | 1.08 (0.83-1.41) |  | 3305 (16.0) | 433 (20.6) | 1.02 (0.81-1.28) |  | 3508 (16.1) | 167 (19.8) | 1.21 (0.81-1.81) |  | 3328 (16.2) | 84 (15.5) | 0.88 (0.52-1.47) |
| **Religious belief** |  |  |  |  |  |  |  |  |  |  |  |  |  |  |  |
| No | 7543 (36.5) | 636 (39.4) | Ref |  | 7593 (36.7) | 788 (37.6) | Ref |  | 7910 (36.2) | 275 (32.5) | Ref |  | 7470 (36.5) | 173 (31.9) | Ref |
| Yes | 11045 (53.4) | 854 (52.9) | 1.19 (0.96-1.49) |  | 11040 (53.4) | 1128 (53.8) | 1.13 (0.93-1.37) |  | 11615 (53.2) | 472 (55.9) | 0.82 (0.59-1.14) |  | 10993 (53.7) | 303 (55.9) | 1.03 (0.67-1.60) |
| **Monthly household income (CNY)** |  |  |  |  |  |  |  |  |  |  |  |  |  |  |  |
| > 5000 | 2893 (14.0) | 228 (14.1) | Ref |  | 2899 (14.0) | 335 (16.0) | Ref |  | 3002 (13.8) | 142 (16.8) | Ref |  | 2813 (13.7) | 68 (12.5) | Ref |
| 3000 - 5000 | 5061 (24.5) | 369 (22.9) | 1.14 (0.91-1.42) |  | 5075 (24.5) | 484 (23.1) | 1.10 (0.91-1.33) |  | 5316 (24.4) | 197 (23.3) | 0.89 (0.65-1.21) |  | 5018 (24.5) | 127 (23.4) | 0.87 (0.57-1.31) |
| ≤ 3000 | 12177 (58.9) | 974 (60.4) | 1.42 (1.15-1.76) ^**^ |  | 12137 (58.7) | 1236 (58.9) | 1.41 (1.18-1.69) ^***^ |  | 12875 (59.0) | 484 (57.3) | 1.09 (0.81-1.46) |  | 12079 (59.0) | 332 (61.3) | 0.95 (0.64-1.41) |
| **Significant negative life events in the last year** |  |  |  |  |  |  |  |  |  |  |  |  |  |  |  |
| No | 18170 (87.8) | 1261 (78.2) | Ref |  | 18221 (88.1) | 1647 (78.5) | Ref |  | 19128 (87.6) | 554 (65.6) | Ref |  | 18001 (87.9) | 341 (62.9) | Ref |
| Yes | 2104 (10.2) | 304 (18.8) | 1.76 (1.48-2.09) ^***^ |  | 2071 (10.0) | 420 (20.0) | 1.97 (1.69-2.30) ^***^ |  | 2213 (10.1) | 274 (32.4) | 2.54 (2.03-3.17) ^***^ |  | 2069 (10.1) | 184 (33.9) | 2.08 (1.55-2.78) ^***^ |
| **Chronic medical condition** |  |  |  |  |  |  |  |  |  |  |  |  |  |  |  |
| No | 17644 (85.3) | 1344 (83.3) | Ref |  | 17674 (85.4) | 1784 (85.1) | Ref |  | 18581 (85.1) | 668 (79.1) | Ref |  | 17486 (85.3) | 427 (78.8) | Ref |
| Yes | 2161 (10.4) | 205 (12.7) | 1.17 (0.97-1.42) |  | 2128 (10.3) | 268 (12.8) | 1.08 (0.91-1.28) |  | 2269 (10.4) | 132 (15.6) | 1.25 (0.96-1.63) |  | 2154 (10.5) | 82 (15.1) | 1.14 (0.81-1.60) |

Note: CI, Confidence interval. CNY, Chinese Yuan. GAD-7, Generalized Anxiety Disorder Scale-7. OR, Odds ratio; SD, Standard deviation.

**^#^**: Multiple imputations were employed. ^ Tibetan, Hui, etc.

* <0.05; ** <0.01, *** <0.001. Statistically significant findings are also marked in bold.

Appendix table 5: The association between intimate partner violence victimization and mental health among the **female** population (Qinghai, China. 2024)

| **Characteristics** | **Anxiety symptoms** | | |  | **Depression symptoms** | | |  | **Suicide ideation** | | |  | **Suicide attempts** | | |
| --- | --- | --- | --- | --- | --- | --- | --- | --- | --- | --- | --- | --- | --- | --- | --- |
|  | **Overall  (N=9725)**  **[n, (%)]** | **GAD-7 score ≥10** | |  | **Overall (N=9668)**  **[n, (%)]** | **PHQ-9 score ≥10** | |  | **Overall (N=10163)**  **[n, (%)]** | **Suicide ideation (Yes)** | |  | **Overall (N=9633)**  **[n, (%)]** | **Suicide attempts (Yes)** | |
|  |  | **(n=782, 8.0)**  **[n, (%)]** | **OR (95% CI) ^#^** |  |  | **(n=1073, 11.1)**  **[n, (%)]** | **OR (95% CI) #** |  |  | **(n=420, 4.1)**  **[n, (%)]** | **OR (95% CI) ^#^** |  |  | **(n=234, 2.4)**  **[n, (%)]** | **OR (95% CI) ^#^** |
| **IPV victimization** |  |  |  |  |  |  |  |  |  |  |  |  |  |  |  |
| No | 8273(89.7) | 648(82.9) | Ref |  | 8641 (89.4) | 873（81.4) | Ref |  | 9059(89.1) | 295(70.2) | Ref |  | 8616 (89.4) | 157 (67.1) | Ref |
| Yes | 651(6.7) | 120(15.3) | **2.01(1.55-2.61)^***^** |  | 658 (6.8%) | 178(16.6) | **2.39(1.91-2.99)^***^** |  | 699(6.9) | 113(26.9) | **4.26(3.18-5.71)^***^** |  | 645 (6.7) | 65 (27.8) | **4.92(3.31-7.30)^***^** |
| **Age years** (Mean, SD) | 38.7(12.5) | 37.7(12.5） | 1.002(0.99-1.01) |  | 38.7 (12.5) | 37.8(12.5) | 1.003(0.99-1.01) |  | 38.7(12.4) | 35.9(11.0) | 0.98(0.97-0.99)^*^ |  | 38.7 (12.4) | 35.6 (10.5) | 0.98(0.96-0.99)^*^ |
| **Living areas** |  |  |  |  |  |  |  |  |  |  |  |  |  |  |  |
| Nonpastoral area | 3122(32.1) | 276(35.3) | Ref |  | 3097(32.0) | 401(37.4) | Ref |  | 3212(31.6) | 111(26.4) | Ref |  | 3101 (32.2) | 40 (17.1) | Ref |
| Pastoral area | 6603(67.9) | 506(64.7) | 1.05(0.86-1.28) |  | 6571(68.0) | 672(62.6) | 0.85(0.72-1.01) |  | 6951(68.4) | 309(73.6) | 1.13(0.86-1.50) |  | 6532 (67.8) | 194 (82.9) | 2.23(1.44-3.47)^***^ |
| **Ethnic group** |  |  |  |  |  |  |  |  |  |  |  |  |  |  |  |
| Han | 3751(38.6) | 322(41.2) | Ref |  | 3744 (38.7) | 429(40.0) | Ref |  | 3884(38.2) | 154(36.7) | Ref |  | 3704 (38.5) | 73 (31.2) | Ref |
| Others | 4841(49.8) | 384(49.1) | 1.03(0.76-1.39) |  | 4764 (49.3) | 549(51.2) | 1.09(0.84-1.41) |  | 5041(49.6) | 208(49.5) | 1.05(0.69-1.58) |  | 4802 (49.8) | 118 (50.4) | 0.76(0.42-1.37) |
| **Marital status** |  |  |  |  |  |  |  |  |  |  |  |  |  |  |  |
| Married | 7511(77.2) | 536(68.5) | Ref |  | 7474 (77.3) | 764(71.2) | Ref |  | 7851(77.3) | 273(65.0) | Ref |  | 7476 (77.6) | 157 (67.1) | Ref |
| Other | 2095(21.5) | 237(30.3) | 1.43(1.17-1.75)**^***^** |  | 2067 (21.4) | 302(28.1) | 1.24(1.03-1.48)**^*^** |  | 2176(21.4) | 141(33.6) | 1.41(1.06-1.86)^*^ |  | 2031 (21.1) | 73 (31.2) | 1.20(0.80-1.78) |
| **Education level** |  |  |  |  |  |  |  |  |  |  |  |  |  |  |  |
| No previous education | 1366(14.0) | 96(12.3) | Ref |  | 1365 (14.1) | 149(13.9) | Ref |  | 1424(14.0) | 43(10.2) | Ref |  | 1345 (14.0) | 28 (12.0) | Ref |
| Primary school | 2718(27.9) | 198(25.3) | 1.14(0.85-1.53) |  | 2679 (27.7) | 243(22.6) | 0.96(0.74-1.25) |  | 2857(28.1) | 97(23.1) | 1.12(0.70-1.79) |  | 2733 (28.4) | 62 (26.5) | 1.34(0.72-2.47) |
| Middle school | 2726(28.0) | 195(24.9) | 0.82(0.59-1.14) |  | 2717 (28.1) | 266(24.8) | 0.79(0.59-1.05) |  | 2862(28.2) | 114(27.1) | 1.13(0.69-1.85) |  | 2699 (28.0) | 69 (29.5) | 1.32(0.68-2.56) |
| College, University or above | 2295(23.6) | 265(33.9) | 1.21(0.81-1.80) |  | 2289 (23.7) | 372(34.7) | 0.99 (0.70-1.41) |  | 2362(23.2) | 134(31.9) | 1.21(0.68-2.16) |  | 2258 (23.4) | 55 (23.5) | 1.23(0.54-2.76) |
| **Occupation** |  |  |  |  |  |  |  |  |  |  |  |  |  |  |  |
| Unemployed | 862(8.9) | 71(9.1) | Ref |  | 828 (8.6) | 122(11.4) | Ref |  | 916(9.0) | 36(8.6) | Ref |  | 860 (8.9) | 23 (9.8) | Ref |
| Farmer/herder | 5497(56.5) | 357(45.7) | 0.86(0.62-1.19) |  | 5483 (56.7) | 430(40.1) | 0.64(0.48-0.85)**^**^** |  | 5744(56.5) | 181(43.1) | 1.21(0.73-2.00) |  | 5427 (56.3) | 116 (49.6) | 1.66(0.83-3.32) |
| Civil servants/Personnel in Public institutions | 1493(15.4) | 150(19.2) | 1.44(0.94-2.22) |  | 1483 (15.3) | 246(22.9) | 1.73(1.20-2.50)**^**^** |  | 1537(15.1) | 92(21.9) | 2.09(1.12-3.90)^*^ |  | 1457 (15.1) | 42 (17.9) | 2.13(0.87-5.20) |
| Other | 1724(17.7) | 186(23.8) | 1.54(1.07-2.23)**^*^** |  | 1725 (17.8) | 258(24.0) | 1.39(1.01-1.90)**^*^** |  | 1799(17.7) | 108(25.7） | 1.83(1.06-3.17)^*^ |  | 1732 (18.0) | 48 (20.5) | 1.73(0.80-3.75) |
| **Religious belief** |  |  |  |  |  |  |  |  |  |  |  |  |  |  |  |
| No | 3754(38.6) | 333(42.6) | Ref |  | 3774 (39.0) | 438(40.8) | Ref |  | 3910(38.5) | 157(37.4) | Ref |  | 3718 (38.6) | 78 (33.3) | Ref |
| Yes | 5057(52.0) | 405(51.8) | 1.05(0.78-1.42) |  | 4995 (51.7) | 550(51.3) | 1.02(0.78-1.32) |  | 5252(51.7) | 229(54.5) | 0.95(0.62-1.43) |  | 5006 (52.0) | 135 (57.7) | 1.24(0.68-2.25) |
| **Monthly household income (CNY)** |  |  |  |  |  |  |  |  |  |  |  |  |  |  |  |
| > 5000 | 1424(14.6) | 126(16.1) | Ref |  | 1417 (14.7) | 184(17.1) | Ref |  | 1462(14.4) | 78(18.6) | Ref |  | 1388 (14.4) | 35 (15.0) | Ref |
| 3000 - 5000 | 2268(23.3) | 158(20.2) | 1.02(0.76-1.37) |  | 2246 (23.2) | 231(21.5) | 1.14(0.89-1.46) |  | 2352(23.1) | 91(21.7) | 0.94(0.63-1.38) |  | 2234 (23.2) | 52 (22.2) | 0.79(0.45-1.40) |
| ≤ 3000 | 5761(59.2) | 473(60.5) | 1.48(1.11-1.96)**^**^** |  | 5739 (59.4) | 637(59.4) | 1.55(1.21-1.98)**^***^** |  | 6050(59.5) | 241(57.4) | 1.08(0.74-1.58) |  | 5732 (59.5) | 142 (60.7) | 1.003(0.58-1.72) |
| **Significant negative life events in the last year** |  |  |  |  |  |  |  |  |  |  |  |  |  |  |  |
| No | 8634(88.8) | 643(82.2) | Ref |  | 8603 (89.0) | 858(80.0) | Ref |  | 9011(88.7) | 308(73.3) | Ref |  | 8556 (88.8) | 166 (70.9) | Ref |
| Yes | 949(9.8) | 129(16.5) | 1.55(1.22-1.97)^***^ |  | 934 (9.7) | 203(18.9) | 1.86(1.51-2.30)**^***^** |  | 985(9.7) | 106(25.2) | 2.21(1.64-2.99)^***^ |  | 924 (9.6) | 63 (26.9) | 1.71(1.11-2.63)^*^ |
| **Chronic medical condition** |  |  |  |  |  |  |  |  |  |  |  |  |  |  |  |
| No | 8481(87.2) | 668(85.4) | Ref |  | 8430 (87.2) | 917(85.5) | Ref |  | 8832(86.9) | 336(80.0) | Ref |  | 8391 (87.1) | 185 (79.1) | Ref |
| Yes | 853(8.8) | 88(11.3) | 1.17(0.88-1.54) |  | 859 (8.9) | 130(12.1) | 1.23(0.96-1.56) |  | 896(8.8) | 63(15.0) | 1.60(1.12-2.28)^**^ |  | 856 (8.9) | 33 (14.1) | 1.44(0.88-2.35) |

Note: CI, Confidence interval. CNY, Chinese Yuan. GAD-7, Generalized Anxiety Disorder Scale-7. OR, Odds ratio; SD, Standard deviation.

**^#^**: Multiple imputations were employed. ^ Tibetan, Hui, etc.

* <0.05; ** <0.01, *** <0.001. Statistically significant findings are also marked in bold.

Appendix table 6: The association between intimate partner violence victimization and mental health among the **male** population (Qinghai, China. 2024)

| **Characteristics** | **Anxiety symptoms** | | |  | **Depression symptoms** | | |  | **Suicide ideation** | | |  | **Suicide attempts** | | |
| --- | --- | --- | --- | --- | --- | --- | --- | --- | --- | --- | --- | --- | --- | --- | --- |
|  | **Overall  (N=10075)**  **[n, (%)]** | **GAD-7 score ≥10** | |  | **Overall (N=10030)**  **[n, (%)]** | **PHQ-9 score ≥10** | |  | **Overall (N=10591)**  **[n, (%)]** | **Suicide ideation (Yes)** | |  | **Overall (N=9881)**  **[n, (%)]** | **Suicide attempts (Yes)** | |
|  |  | **(n=745, 7.4)**  **[n, (%)]** | **OR (95% CI) ^#^** |  |  | **(n=939, 9.4)**  **[n, (%)]** | **OR (95% CI) ^#^** |  |  | **(n=363, 3.4)**  **[n, (%)]** | **OR (95% CI) ^#^** |  |  | **(n=260, 2.6)**  **[n, (%)]** | **OR (95% CI) ^#^** |
| **IPV victimization** |  |  |  |  |  |  |  |  |  |  |  |  |  |  |  |
| No | 9124(90.6) | 584(78.4) | Ref |  | 9083 (90.6) | 783 (83.4) | Ref |  | 9536 (90.0) | 225 (62.0) | Ref |  | 8944 (90.5) | 163 (62.7) | Ref |
| Yes | 535(5.3) | 115(15.4) | **3.25(2.42-4.38)^***^** |  | 531 (5.3) | 128 (13.6) | **2.68(2.03-3.54)^***^** |  | 580 (5.5) | 123 (33.9) | **7.05(4.93-10.08)^***^** |  | 521 (5.3) | 87 (33.5) | **6.83(4.43-10.53)^***^** |
| **Age years** (Mean, SD) | 41.9(12.6) | 40.9(13.4) | 0.99(0.98-0.99)^**^ |  | 41.9 (12.7) | 40.7 (13.2) | 0.99(0.98-0.99)^*^ |  | 41.9 (12.6) | 41.0 (12.8) | 0.99(0.98-1.01) |  | 41.9 (12.7) | 40.5 (13.0) | 0.99(0.98-1.01) |
| **Living areas** |  |  |  |  |  |  |  |  |  |  |  |  |  |  |  |
| Nonpastoral area | 2970(29.5) | 211 (28.3) | Ref |  | 2931 (29.2) | 304 (32.4) | Ref |  | 3073 (29.0) | 66 (18.2) | Ref |  | 2947 (29.8) | 29 (11.2) | Ref |
| Pastoral area | 7105(70.5) | 534 (71.7) | 1.13(0.90-1.43) |  | 7099 (70.8) | 635 (67.6) | 0.77(0.63-0.93)^**^ |  | 7518 (71.0) | 297 (81.8) | 1.34(0.93-1.92) |  | 6934 (70.2) | 231 (88.8) | 2.83(1.73-4.64)**^***^** |
| **Ethnic group** |  |  |  |  |  |  |  |  |  |  |  |  |  |  |  |
| Han | 3552(35.3) | 291 (39.1) | Ref |  | 3558 (35.5) | 339 (36.1) | Ref |  | 3733 (35.2) | 106 (29.2) | Ref |  | 3526 (35.7) | 73 (28.1) | Ref |
| Others ^ | 5092 (50.5) | 342 (45.9) | 0.54(0.39-0.76)^***^ |  | 5025 (50.1) | 463 (49.3) | 0.80(0.60-1.06) |  | 5320 (50.2) | 189 (52.1) | 0.98(0.57-1.70) |  | 4987 (50.5) | 137 (52.7) | 0.60(0.31-1.16) |
| **Marital status** |  |  |  |  |  |  |  |  |  |  |  |  |  |  |  |
| Married | 7923 (78.6) | 525 (70.5) | Ref |  | 7917 (78.9) | 677 (72.1) | Ref |  | 8317 (78.5) | 245 (67.5) | Ref |  | 7813 (79.1) | 185 (71.2) | Ref |
| Other | 1926 (19.1) | 198 (26.6) | 1.34(1.06-1.69)^*^ |  | 1882 (18.8) | 241 (25.7) | 1.24(1.01-1.53)^*^ |  | 2022 (19.1) | 111 (30.6) | 1.49(1.06-2.09)^*^ |  | 1838 (18.6) | 68 (26.2) | 0.93(0.60-1.45) |
| **Education level** |  |  |  |  |  |  |  |  |  |  |  |  |  |  |  |
| No previous education | 1199 (11.9) | 89 (11.9) | Ref |  | 1208 (12.0) | 120 (12.8) | Ref |  | 1260 (11.9) | 43 (11.8) | Ref |  | 1176 (11.9) | 34 (13.1) | Ref |
| Primary school | 3209 (31.9) | 280 (37.6) | 1.39(1.01-1.91)^*^ |  | 3117 (31.1) | 308 (32.8) | 1.23(0.93-1.63) |  | 3350 (31.6) | 128 (35.3) | 1.55(0.95-2.50) |  | 3104 (31.4) | 108 (41.5) | 1.59(0.94-2.70) |
| Middle school | 3507 (34.8) | 210 (28.2) | 0.71(0.50-1.02) |  | 3515 (35.0) | 275 (29.3) | 0.84(0.62-1.15) |  | 3697 (34.9) | 78 (21.5) | 0.75(0.43-1.29) |  | 3481 (35.2) | 55 (21.2) | 0.65(0.35-1.20) |
| College, University or above | 1424 (14.1) | 121 (16.2) | 0.90(0.57-1.42) |  | 1443 (14.4) | 183 (1.95) | 1.16(0.78-1.73) |  | 1493 (14.1) | 74 (20.4) | 1.56(0.79-3.09) |  | 1414 (14.3) | 43 (16.5) | 1.48(0.66-3.32) |
| **Occupation** |  |  |  |  |  |  |  |  |  |  |  |  |  |  |  |
| Unemployed | 750 (7.4) | 87 (11.7) | Ref |  | 742 (7.4) | 110 (11.7) | Ref |  | 804 (7.6) | 41 (11.3) | Ref |  | 739 (7.5) | 32 (12.3) | Ref |
| Farmer/herder | 6717 (66.7) | 442 (59.3) | 0.61(0.44-0.84)^**^ |  | 6665 (66.5) | 535 (57.0) | 0.55(0.41-0.74)^***^ |  | 7011 (66.2) | 222 (61.2) | 0.73(0.44-1.20) |  | 6566 (66.5) | 180 (69.2) | 0.68(0.39-1.18) |
| Civil servants/Personnel in Public institutions | 996 (9.9) | 93 (12.5) | 1.04(0.64-1.67) |  | 1015 (10.1) | 125 (13.3) | 0.78(0.51-1.18) |  | 1044 (9.9) | 43 (11.8) | 0.69(0.33-1.44) |  | 961 (9.7) | 16 (6.2) | 0.24(0.09-0.66)**^**^** |
| Other | 1465 (14.5) | 116 (15.6) | 0.64(0.43-0.96)^*^ |  | 1449 (14.4) | 159 (16.9) | 0.70(0.50-0.99)^*^ |  | 1560 (14.7) | 50 (13.8) | 0.65(0.35-1.22) |  | 1457 (14.7) | 30 (11.5) | 0.45(0.21-0.94)**^*^** |
| **Religious belief** |  |  |  |  |  |  |  |  |  |  |  |  |  |  |  |
| No | 3538 (35.1) | 281 (37.7) | Ref |  | 3568 (35.6) | 318 (33.9) | Ref |  | 3728 (35.2) | 111 (30.6) | Ref |  | 3506 (35.5) | 80 (30.8) | Ref |
| Yes | 5514 (54.7) | 405 (54.4) | 1.40(1.02-1.94)^*^ |  | 5474 (54.6) | 538 (57.3) | 1.30(0.98-1.73) |  | 5755 (54.3) | 199 (54.8) | 0.68(0.40-1.17) |  | 5425 (54.9) | 141 (54.2) | 0.89(0.46-1.71) |
| **Monthly household income (CNY)** |  |  |  |  |  |  |  |  |  |  |  |  |  |  |  |
| > 5000 | 1367 (13.6) | 94 (12.6) | Ref |  | 1374 (13.7) | 136 (14.5) | Ref |  | 1424 (13.4) | 52 (14.3) | Ref |  | 1323 (13.4) | 29 (11.2) | Ref |
| 3000 - 5000 | 2601 (25.8) | 190 (25.5) | 1.27(0.91-1.78) |  | 2639 (26.3) | 230 (24.5) | 1.05(0.79-1.39) |  | 2754 (26.0) | 86 (23.7) | 0.80(0.48-1.33) |  | 2595 (26.3) | 63 (24.2) | 0.89(0.48-1.66) |
| ≤ 3000 | 5892 (58.5) | 449 (60.3) | 1.35(0.97-1.87) |  | 5793 (57.8) | 557 (59.3) | 1.28(0.97-1.68) |  | 6169 (58.2) | 216 (59.5) | 1.05(0.65-1.69) |  | 5740 (58.1) | 160 (61.5) | 0.87(0.49-1.56) |
| **Significant negative life events in the last year** |  |  |  |  |  |  |  |  |  |  |  |  |  |  |  |
| No | 8808 (87.4) | 559 (75.0) | Ref |  | 8795 (87.7) | 725 (77.2) | Ref |  | 9231 (87.2) | 215 (59.2) | Ref |  | 8631 (87.3) | 157 (60.4) | Ref |
| Yes | 1054 (10.5) | 154 (20.7) | 1.91(1.48-2.47)^***^ |  | 1039 (10.4) | 201 (21.4) | 2.03(1.62-2.55)^***^ |  | 1115 (10.5) | 140 (38.6) | 2.89(2.05-4.07)^***^ |  | 1041 (10.5) | 99 (38.1) | 2.44(1.61-3.70)^***^ |
| **Chronic medical condition** |  |  |  |  |  |  |  |  |  |  |  |  |  |  |  |
| No | 8425 (83.6) | 608 (81.6) | Ref |  | 8404 (83.8) | 790 (84.1) | Ref |  | 8843 (83.5) | 279 (76.9) | Ref |  | 8261 (83.6) | 202 (77.7) | Ref |
| Yes | 1221 (12.1) | 105 (14.1) | 1.19(0.91-1.56) |  | 1186 (11.8) | 131 (14.0) | 0.96(0.75-1.24) |  | 1276 (12.0) | 63 (17.4) | 0.99(0.67-1.49) |  | 1211 (12.3) | 45 (17.3) | 0.98(0.61-1.58) |

Note: CI, Confidence interval. CNY, Chinese Yuan. GAD-7, Generalized Anxiety Disorder Scale-7. OR, Odds ratio; SD, Standard deviation.

**^#^**: Multiple imputations were employed. ^ Tibetan, Hui, etc.

* <0.05; ** <0.01, *** <0.001. Statistically significant findings are also marked in bold.

Appendix Table 7: Comparison of characteristics among **female and male participants** between those with and without intimate partner violence victimization **after optimal full matching** (Qinghai, China. 2024)

|  | **Female** participants **after**  matching  (N=7029) | |  | **Male** participants **after**  matching  (N=6907) | | |
| --- | --- | --- | --- | --- | --- | --- |
|  | **Intimate Partner Violence** | |  | **Intimate Partner Violence** | | |
|  | None  (N =6501) | Any  (N = 528) |  | None  (N =6543) | Any  (N = 364) | |
| **Age years** |  |  |  |  |  |  |
| Mean (SD) | 38.4 (12.4) | 37.2 (10.2) |  | 41.9 (12.7) | 41.9 (12.7) |  |
| Missing | - | - |  | - | - |  |
| **Living areas** |  |  |  |  |  |  |
| Pastoral area | 4165 (64.1) | 352 (66.7) |  | 4359 (66.6) | 251 (69.0) |  |
| Nonpastoral area | 2336 (35.9) | 176 (33.3) |  | 2184 (33.4) | 113 (31.0) |  |
| **Ethnic group** |  |  |  |  |  |  |
| Han | 2994 (46.1) | 191 (36.2) |  | 2917 (44.6) | 119 (32.7) |  |
| Others ^ (Tibetan, Hui, etc.) | 3507 (53.9) | 337 (63.8) |  | 3626 (55.4) | 245 (67.3) |  |
| Missing | - | - |  | - | - |  |
| **Marital status** |  |  |  |  |  |  |
| Married | 5091 (78.3) | 398 (75.4) |  | 5297 (81.0) | 274 (75.3) |  |
| Other | 1410 (21.7) | 130 (24.6) |  | 1246 (19.0) | 90 (24.7) |  |
| Missing | - | - |  | - | - |  |
| **Education level** |  |  |  |  |  |  |
| No previous education | 896 (13.8) | 78 (14.8) |  | 800 (12.2) | 60 (16.5) |  |
| Primary school | 1832 (28.2) | 126 (23.9) |  | 2047 (31.3) | 121 (33.2) |  |
| Middle school | 1908 (29.3) | 182 (34.5) |  | 2607 (39.8) | 114 (31.3) |  |
| College, University or above | 1865 (28.7) | 142 (26.9) |  | 1089 (16.6) | 69 (19.0) |  |
| Missing | - | - |  | - | - |  |
| **Occupation** |  |  |  |  |  |  |
| Unemployed | 493 (7.6) | 77 (14.6) |  | 416 (6.4) | 36 (9.9) |  |
| Farmer/herder | 3554 (54.7) | 232 (43.9) |  | 4293 (65.6) | 225 (61.8) |  |
| Civil servants/Personnel in public institutions | 1194 (18.4) | 107 (20.3) |  | 790 (12.1) | 48 (13.2) |  |
| Other | 1260 (19.4) | 112 (21.2) |  | 1044 (16.0) | 55 (15.1) |  |
| Missing | - | - |  | - | - |  |
| **Religious belief** |  |  |  |  |  |  |
| No | 2862 (44.0) | 176 (33.3) |  | 2712 (41.4) | 102 (28.0) |  |
| Yes | 3639 (56.0) | 352 (66.7) |  | 3831 (58.6) | 262 (72.0) |  |
| Missing | - | - |  | - | - |  |
| **Monthly household income (CNY)** |  |  |  |  |  |  |
| ≤ 3000 | 3821 (58.8) | 323 (61.2) |  | 3741 (57.2) | 225 (61.8) |  |
| 3000 - 5000 | 1539 (23.7) | 120 (22.7) |  | 1792 (27.4) | 72 (19.8) |  |
| > 5000 | 1141 (17.6) | 85 (16.1) |  | 1010 (15.4) | 67 (18.4) |  |
| Missing | - | - |  | - | - |  |
| **Significant negative life events in the last year** |  |  |  |  |  |  |
| No | 5938 (91.3) | 381 (72.2) |  | 5963 (91.1) | 210 (57.7) |  |
| Yes | 563 (8.7) | 147 (27.8) |  | 580 (8.9) | 154 (42.3) |  |
| Missing | - | - |  | - | - |  |
| **Chronic medical condition** |  |  |  |  |  |  |
| No | 5915 (91.0) | 428 (81.1) |  | 5749 (87.9) | 263 (72.3) |  |
| Yes | 586 (9.0) | 100 (18.9) |  | 794 (12.1) | 101 (27.7) |  |
| Missing | - | - |  | - | - |  |

^: Other ethnic group includes Tibetan, Hui, etc.

* Wilcoxon rank sum test; Pearson’s Chi-squared test

Note: CNY, Chinese Yuan; SD, Standard deviation.

Appendix table 8: Associations between intimate partner violence victimization and mental health problems using propensity score matching among the **whole participants (N=13969)** (Qinghai, China. 2024)

|  | **Anxiety symptoms** | | |
| --- | --- | --- | --- |
|  | Overall  (N=13488) | **GAD-7 score ≥10** | |
|  |  | Yes (n=1107)  n (%) | OR (95% CI) |
| **IPV** (No) | 12643 (93.7) | 942 (85.1) | Ref |
| **IPV** (Yes) | 845 (6.3) | 165 (14.9) | | 2.45 (2.01-2.96) ^***^ | | --- | |
|  | **Depression symptoms** | | |
|  | Overall  (N=13425) | **PHQ-9 score ≥10** | |
|  |  | Yes (n=1491)  n (%) | OR (95% CI) |
| **IPV** (No) | 12584 (93.7) | 1270 (85.2) | Ref |
| **IPV** (Yes) | 841 (6.3) | 221 (14.8) | 2.50 (2.10-2.97) ^***^ |
|  | **Suicide behaviors** | | |
|  | Overall  (N=13936) | **Suicide ideation** | |
|  |  | Yes (n=502)  n (%) | OR (95% CI) |
| **IPV** (No) | 13044 (93.6) | 352 (70.1) | Ref |
| **IPV** (Yes) | 892 (6.4) | 150 (29.9) | 5.38 (4.30-6.71) ^***^ |
|  | **Suicide behaviors** | | |
|  | Overall  (N=13330) | **Suicide attempts** | |
|  |  | Yes (n=292)  n (%) | OR (95% CI) |
| **IPV** (No) | 12491 (93.7) | 203 (69.5) | Ref |
| **IPV** (Yes) | 839 (6.3) | 89 (30.5) | 5.84 (4.36-7.76) ^***^ |

Note: GAD-7, Generalized Anxiety Disorder Scale-7; PHQ-9, Patient Health Questionnaire-9.

SD: Standard deviation.

Appendix table 9: Associations between intimate partner violence victimization and mental health problems using propensity score matching among the **female participants (N=7029)** (Qinghai, China. 2024)

|  | **Anxiety symptoms** | | |
| --- | --- | --- | --- |
|  | Overall  (N=6826) | **GAD-7 score ≥10** | |
|  |  | Yes (n=615)  n (%) | OR (95% CI) |
| **IPV** (No) | 6323 (92.6) | 529 (86.0) | Ref |
| **IPV** (Yes) | 503 (7.4) | 86 (14.0) | | 2.01 (1.54-2.59) ^***^ | | --- | |
|  | **Depression symptoms** | | |
|  | Overall  (N=6780) | **PHQ-9 score ≥10** | |
|  |  | Yes (n=832)  n (%) | OR (95% CI) |
| **IPV** (No) | 6275 (92.6) | 699 (84.0) | Ref |
| **IPV** (Yes) | 505 (7.4) | 133 (16.0) | 2.39 (1.90-2.98) ^***^ |
|  | **Suicide behaviors** | | |
|  | Overall  (N=7029) | **Suicide ideation** | |
|  |  | Yes (n=293)  n (%) | OR (95% CI) |
| **IPV** (No) | 6501 (92.5) | 213 (72.7) | Ref |
| **IPV** (Yes) | 528 (7.5) | 80 (27.3) | 4.26 (3.16-5.68) ^***^ |
|  | **Suicide behaviors** | | |
|  | Overall  (N=6752) | **Suicide attempts** | |
|  |  | Yes (n=145)  n (%) | OR (95% CI) |
| **IPV** (No) | 6252 (92.6) | 102 (70.3) | Ref |
| **IPV** (Yes) | 500 (7.4) | 43 (29.7) | 4.92 (3.28-7.25) ^***^ |

Note: GAD-7, Generalized Anxiety Disorder Scale-7; PHQ-9, Patient Health Questionnaire-9.

SD: Standard deviation.

**Appendix table 10: Associations between intimate partner violence victimization and mental health problems using propensity score matching among the male participants (N=6907) (Qinghai, China. 2024)**

|  | **Anxiety symptoms** | | |
| --- | --- | --- | --- |
|  | Overall  (N=6662) | **GAD-7 score ≥10** | |
|  |  | Yes (n=492)  n (%) | OR (95% CI) |
| **IPV** (No) | 6320 (94.9) | 413 (83.9) | Ref |
| **IPV** (Yes) | 342 (5.1) | 79 (16.1) | 3.25 (2.41-4.36) ^***^ |
|  | **Depression symptoms** | | |
|  | Overall  (N=6645) | **PHQ-9 score ≥10** | |
|  |  | Yes (n=659)  n (%) | OR (95% CI) |
| **IPV** (No) | 6309 (94.9) | 571 (86.6) | Ref |
| **IPV** (Yes) | 336 (5.1) | 88 (13.4) | 2.68 (2.02-3.53) ^***^ |
|  | **Suicide behaviors** | | |
|  | Overall  (N=6645) | **Suicide ideation** | |
|  |  | Yes (n=659)  n (%) | OR (95% CI) |
| **IPV** (No) | 6309 (94.9) | 571 (86.6) | Ref |
| **IPV** (Yes) | 336 (5.1) | 88 (13.4) | 7.05 (4.92-10.05) ^***^ |
|  | **Suicide behaviors** | | |
|  | Overall  (N=6578) | **Suicide attempts** | |
|  |  | Yes (n=147)  n (%) | OR (95% CI) |
| **IPV** (No) | 6239 (94.8) | 101 (68.7) | Ref |
| **IPV** (Yes) | 339 (5.2) | 46 (31.3) | 6.83 (4.40-10.47) ^***^ |

Note: GAD-7, Generalized Anxiety Disorder Scale-7; PHQ-9, Patient Health Questionnaire-9.

SD: Standard deviation.

Appendix table 11: Gender-stratified associations between intimate partner violence victimization and **possible anxiety** by each characteristic (Qinghai, China. 2024)

|  | Female (N=10163) | | |  | Male | | |
| --- | --- | --- | --- | --- | --- | --- | --- |
|  | % (N_IPV_  / N_Overall)_ | GAD-7 score ≥10 | |  | % (N_IPV_  / N_Overall)_ | GAD-7 score ≥10 | |
|  |  | % (N_IPV_  / N_Overall)_ | OR (95% CI) |  |  | % (N_IPV_  / N_Overall)_ | OR (95% CI) |
| **By age groups (years)** |  |  |  |  |  |  |  |
| < 35 | 7.1 (295/4128) | 17.4 (63/362) | **2.37 (1.63-3.44)** |  | 5.5 (168/3034) | 17.3 (47/271) | **4.44 (2.73-7.23)** |
| 35-45 | 7.3 (191/2608) | 16.4 (34/207) | **2.23 (1.38-3.58)** |  | 4.7 (133/2817) | 12.1 (22/182) | **2.48 (1.30-4.73)** |
| 45-55 | 6.8 (123/1797) | 14.7 (19/129) | 1.91 (0.93-3.92) |  | 5.5 (135/2453) | 16.9 (27/160) | **4.10 (2.16-7.78)** |
| 55 or more | 3.6 (40/1121) | 4.9 (4/82) | - |  | 5.1 (85/1670) | 13.6 (17/125) | 2.20 (0.99-4.87) |
| **By living areas** |  |  |  |  |  |  |  |
| Pastoral area | 6.7 (444/6603) | 15.0 (76/506) | **1.81 (1.30-2.52)** |  | 5.5 (394/7105) | 15.9 (85/534) | **2.93 (2.02-4.25)** |
| Nonpastoral area | 6.6 (207/3122) | 15.9 (44/276) | **2.25 (1.46-3.47)** |  | 4.7 (141/2970) | 14.2 (30/211) | **4.13 (2.49-6.85)** |
| **By ethnic group** |  |  |  |  |  |  |  |
| Han | 5.6 (209/3751) | 13.0 (42/322) | **2.16 (1.41-3.30)** |  | 4.1 (146/3552) | 7.9 (23/291) | **2.24 (1.31-3.83)** |
| Others | 7.7 (374/4841) | 15.4 (59/384) | **1.84 (1.31-2.57)** |  | 5.8 (297/5092) | 19.6 (67/342) | **3.90 (2.71-5.61)** |
| **By marital status** |  |  |  |  |  |  |  |
| Married | 6.6 (496/7511) | 16.4 (88/536) | **2.31 (1.70-3.13)** |  | 4.8 (384/7923) | 14.7 (77/525) | **2.99 (2.10-4.25)** |
| Other | 7.2 (150/2095) | 13.1 (31/237) | 1.41 (0.84-2.37) |  | 7.1 (137/1926) | 18.7 (37/198) | **4.27 (2.38-7.67)** |
| **By education level** |  |  |  |  |  |  |  |
| No previous education | 7.5 (103/1366) | 13.5 (13/96) | 1.29 (0.58-2.88) |  | 6.4 (77/1199) | 19.1 (17/89) | 1.97 (0.83-4.69) |
| Primary school | 5.8 (158/2718) | 14.1 (28/198) | **1.94 (1.12-3.34)** |  | 5.2 (166/3209) | 16.1 (45/280) | **3.75 (2.26-6.22)** |
| Middle school | 8.0 (217/2726) | 18.5 (36/195) | **2.12 (1.29-3.47)** |  | 4.4 (154/3507) | 11.9 (25/210) | **3.15 (1.76-5.61)** |
| College, University or above | 6.4 (147/2295) | 14.7 (39/265) | **2.23 (1.44-3.47)** |  | 5.9 (84/1424) | 19.8 (24/121) | **3.42 (1.80-6.52)** |
| **By occupation** |  |  |  |  |  |  |  |
| Unemployed | 10.6 (91/862) | 8.5 (6/71) | 0.89 (0.34-2.33) |  | 6.5 (49/750) | 12.6 (11/87) | **2.86 (1.13-7.25)** |
| Farmer/herder | 5.6 (308/5497) | 15.1 (54/357) | **2.17 (1.44-3.28)** |  | 5.2 (349/6717) | 17.2 (76/442) | **3.18 (2.15-4.71)** |
| Civil servants/Personnel in public institutions | 7.7 (115/1493) | 16.0 (24/150) | **1.86 (1.07-3.24)** |  | 6.0 (60/996) | 14.0 (13/93) | **3.41 (1.58-7.34)** |
| Other | 7.4 (128/1724) | 17.2 (32/186) | **2.88 (1.73-4.81)** |  | 4.8 (70/1465) | 12.9 (15/116) | **4.18 (1.85-9.44)** |
| **By religious belief** |  |  |  |  |  |  |  |
| No | 5.5 (206/3754) | 13.5 (45/333) | **2.17 (1.41-3.36)** |  | 4.1 (144/3538) | 8.9 (25/281) | 1.65 (0.88-3.12) |
| Yes | 8.0 (407/5057) | 17.0 (69/405) | **1.84 (1.32-2.56)** |  | 6.1 (339/5514) | 20.7 (84/405) | **4.14 (2.93-5.85)** |
| **By monthly household income** |  |  |  |  |  |  |  |
| ≤ 3000 CNY | 6.9 (398/5761) | 14.8 (70/473) | **1.87 (1.33-2.62)** |  | 5.5 (326/5892) | 16.9 (76/449) | **3.10 (2.10-4.55)** |
| 3000 - 5000 CNY | 6.7 (152/2268) | 15.8 (25/158) | 1.76 (0.97-3.21) |  | 4.6 (119/2601) | 13.7 (26/190) | **3.18 (1.62-6.24)** |
| > 5000 CNY | 6.1 (87/1424) | 15.9 (20/126) | **3.13 (1.72-5.67)** |  | 5.6 (77/1367) | 13.8 (13/94) | **4.12 (2.02-8.39)** |
| **By significant negative life events** |  |  |  |  |  |  |  |
| No | 5.4 (465/8634) | 9.8 (63/643) | **1.68 (1.21-2.33)** |  | 3.3 (289/8808) | 7.3 (41/559) | **2.98 (2.00-4.46)** |
| Yes | 18.9 (179/949) | 41.9 (54/129) | **3.18 (1.93-5.23)** |  | 21.9 (231/1054) | 46.8 (72/154) | **3.67 (2.27-5.93)** |
| **By chronic medical condition** |  |  |  |  |  |  |  |
| No | 6.0 (513/8481) | 13.9 (93/668) | **2.06 (1.54-2.75)** |  | 4.1 (348/8425) | 13.0 (79/608) | **3.46 (2.44-4.89)** |
| Yes | 14.2 (121/853) | 28.4 (25/88) | 1.78 (0.93-3.39) |  | 12.0 (146/1221) | 30.5 (32/105) | **3.01 (1.65-5.49)** |

CNY, Chinese Yuan; PHQ-9, Patient Health Questionnaire-9

Appendix table 12: Gender-stratified associations between intimate partner violence victimization and **possible depression** by each characteristic (Qinghai, China. 2024)

|  | Female | | |  | Male | | |
| --- | --- | --- | --- | --- | --- | --- | --- |
|  | % (N_IPV_  / N_Overall)_ | PHQ-9 score ≥10 | |  | % (N_IPV_  / N_Overall)_ | PHQ-9 score ≥10 | |
|  |  | % (N_IPV_  / N_Overall)_ | OR (95% CI) |  |  | % (N_IPV_  / N_Overall)_ | OR (95% CI) |
| **By age groups (years)** |  |  |  |  |  |  |  |
| < 35 | 7.2 (295/4090) | 16.5 (80/486) | **2.24 (1.60-3.14)** |  | 5.7 (172/3029) | 14.0 (48/342) | **2.78 (1.73-4.46)** |
| 35-45 | 7.7 (198/2588) | 18.0 (53/295) | **2.71 (1.80-4.08)** |  | 4.7 (132/2809) | 10.9 (27/247) | **2.88 (1.64-5.05)** |
| 45-55 | 7.0 (125/1796) | 21.2 (38/179) | **3.20 (1.85-5.52)** |  | 5.5 (133/2404) | 16.8 (32/190) | **3.38 (1.89-6.05)** |
| 55 or more | 3.4 (38/1121) | 6.6 (7/106) | 0.76 (0.21-2.84) |  | 4.8 (80/1683) | 12.5 (19/152) | 1.63 (0.75-3.55) |
| **By living areas** |  |  |  |  |  |  |  |
| Pastoral area | 6.9 (455/6571) | 16.1 (108/672) | **2.15 (1.61-2.88)** |  | 5.6 (395/7099) | 15.6 (99/635) | **2.76 (1.94-3.91)** |
| Nonpastoral area | 6.6 (203/3097) | 17.5 (70/401) | **2.78 (1.92-4.03)** |  | 4.6 (136/2931) | 9.5 (29/304) | **2.56 (1.58-4.17)** |
| **By ethnic group** |  |  |  |  |  |  |  |
| Han | 5.5 (206/3744) | 13.1 (56/429) | **2.35 (1.63-3.38)** |  | 4.0 (141/3558) | 8.0 (27/339) | **2.12 (1.29-3.47)** |
| Others | 8.0 (380/4764) | 17.9 (98/549) | **2.31 (1.73-3.10)** |  | 6.0 (302/5025) | 15.8 (73/463) | **3.04 (2.15-4.30)** |
| **By marital status** |  |  |  |  |  |  |  |
| Married | 6.6 (494/7474) | 17.1 (131/764) | **2.63 (2.03-3.41)** |  | 4.9 (385/7917) | 13.0 (88/677) | **2.63 (1.90-3.64)** |
| Other | 7.6 (158/2067) | 15.2 (46/302) | **1.61 (1.01-2.56)** |  | 7.1 (134/1882) | 15.8 (38/241) | **2.71 (1.54-4.76)** |
| **By education level** |  |  |  |  |  |  |  |
| No previous education | 7.5 (102/1365) | 19.5 (29/149) | **3.43 (1.86-6.33)** |  | 6.9 (83/1208) | 17.5 (21/120) | **2.38 (1.13-5.03)** |
| Primary school | 5.7 (153/2679) | 13.2 (32/243) | 1.49 (0.87-2.57) |  | 4.9 (153/3117) | 14.6 (45/308) | **3.11 (1.88-5.14)** |
| Middle school | 8.1 (219/2717) | 18.0 (48/266) | **1.98 (1.29-3.03)** |  | 4.3 (152/3515) | 9.5 (26/275) | **2.18 (1.27-3.72)** |
| College, University or above | 6.8 (155/2289) | 16.9 (63/372) | **3.07 (2.11-4.46)** |  | 6.0 (87/1443) | 18.0 (33/183) | **3.10 (1.72-5.58)** |
| **By occupation** |  |  |  |  |  |  |  |
| Unemployed | 10.6 (88/828) | 13.1 (16/122) | 1.62 (0.78-3.39) |  | 6.7 (50/742) | 16.4 (18/110) | **3.29 (1.42-7.64)** |
| Farmer/herder | 5.7 (314/5483) | 16.7 (72/430) | **2.37 (1.63-3.43)** |  | 5.1 (338/6665) | 14.0 (75/535) | **2.47 (1.70-3.59)** |
| Civil servants/Personnel in public institutions | 8.3 (123/1483) | 18.7 (46/246) | **2.98 (1.93-4.60)** |  | 5.8 (59/1015) | 11.2 (14/125) | **2.46 (1.15-5.26)** |
| Other | 7.3 (126/1725) | 15.5 (40/258) | **2.46 (1.53-3.95)** |  | 5.2 (75/1449) | 12.6 (20/159) | **3.06 (1.55-6.06)** |
| **By religious belief** |  |  |  |  |  |  |  |
| No | 5.4 (203/3774) | 13.5 (59/438) | **2.17 (1.48-3.18)** |  | 4.0 (142/3568) | 9.1 (29/318) | **2.02 (1.16-3.51)** |
| Yes | 8.2 (412/4995) | 19.5(107/550) | **2.50 (1.88-3.32)** |  | 6.2 (339/5474) | 17.3 (93/538) | **2.97 (2.13-4.13)** |
| **By monthly household income** |  |  |  |  |  |  |  |
| ≤ 3000 CNY | 7.0 (402/5739) | 16.0 (102/637) | **1.98 (1.46-2.68)** |  | 5.5 (320/5793) | 15.6 (87/557) | **2.68 (1.86-3.84)** |
| 3000 - 5000 CNY | 6.9 (154/2246) | 20.3 (47/231) | **3.33 (2.10-5.29)** |  | 4.5 (120/2639) | 10.9 (25/230) | **2.22 (1.13-4.36)** |
| > 5000 CNY | 6.4 (91/1417) | 15.2 (28/184) | **2.79 (1.67-4.66)** |  | 5.7 (78/1374) | 11.0 (15/136) | **2.90 (1.51-5.76)** |
| **By significant negative life events** |  |  |  |  |  |  |  |
| No | 5.4 (468/8603) | 11.4 (98/858) | **2.15 (1.64-2.82)** |  | 3.3 (288/8795) | 7.3 (53/725) | **2.87 (2.00-4.12)** |
| Yes | 19.6 (183/934) | 38.4 (78/203) | **3.05 (1.97-4.73)** |  | 21.9 (228/1039) | 35.8 (72/201) | **2.21 (1.39-3.50)** |
| **By chronic medical condition** |  |  |  |  |  |  |  |
| No | 6.1 (513/8430) | 14.3 (131/917) | **2.35 (1.83-3.01)** |  | 4.1 (346/8404) | 11.1 (88/790) | **2.76 (2.00-3.80)** |
| Yes | 14.6 (125/859) | 32.3 (42/130) | **2.63 (1.53-4.51)** |  | 12.1 (143/1186) | 26.0 (34/131) | **2.57 (1.42-4.63)** |

CNY, Chinese Yuan; PHQ-9, Patient Health Questionnaire-9

Appendix table 13: Gender-stratified associations between intimate partner violence victimization and **suicide ideation** by each characteristic (Qinghai, China. 2024)

|  | Female | | |  | Male | | |
| --- | --- | --- | --- | --- | --- | --- | --- |
|  | % (N_IPV_  / N_Overall)_ | PHQ-9 score ≥10 | |  | % (N_IPV_  / N_Overall)_ | PHQ-9 score ≥10 | |
|  |  | % (N_IPV_  / N_Overall)_ | OR (95% CI) |  |  | % (N_IPV_  / N_Overall)_ | OR (95% CI) |
| **By age groups (years)** |  |  |  |  |  |  |  |
| < 35 | 7.3 (312/4290) | 24.4 (51/209) | **2.80 (1.80-4.35)** |  | 5.9 (188/3204) | 32.3 (41/127) | **5.34 (2.82-10.12)** |
| 35-45 | 7.6 (208/2753) | 28.0 (30/107) | **4.92 (2.84-8.54)** |  | 4.8 (143/2972) | 32.7 (32/98) | **9.60 (4.92-18.75)** |
| 45-55 | 7.2 (135/1880) | 33.8 (24/71) | **7.00 (3.29-14.91)** |  | 5.7 (146/2559) | 40.3 (31/77) | **12.07 (5.50-26.52)** |
| 55 or more | 3.6 (42/1162) | 28.0 (7/25) | **8.82 (2.59-30.03)** |  | 5.1 (89/1747) | 30.4 (17/56) | **5.09 (1.84-14.10)** |
| **By living areas** |  |  |  |  |  |  |  |
| Pastoral area | 6.9 (482/6951) | 27.8 (86/309) | **4.66 (3.26-6.65)** |  | 5.7 (432/7518) | 35.7 (106/297) | **6.88 (4.47-10.59)** |
| Nonpastoral area | 6.8 (217/3212) | 24.3 (27/111) | **3.52 (2.06-6.01)** |  | 4.8 (148/3073) | 25.8 (17/66) | **6.85 (3.42-13.73)** |
| **By ethnic group** |  |  |  |  |  |  |  |
| Han | 5.7 (221/3884) | 16.9 (26/154) | **2.90 (1.74-4.85)** |  | 4.1 (153/3733) | 19.8 (21/106) | **4.42 (2.30-8.51)** |
| Others | 7.9 (400/5041) | 31.7 (66/208) | **5.13 (3.54-7.44)** |  | 6.1 (327/5320) | 39.7 (75/189) | **8.53 (5.42-13.42)** |
| **By marital status** |  |  |  |  |  |  |  |
| Married | 6.7 (527/7851) | 27.1 (74/273) | **4.76 (3.34-6.80)** |  | 5.0 (415/8317) | 28.2 (69/245) | **6.49 (4.17-10.11)** |
| Other | 7.6 (165/2176) | 27.0 (38/141) | **3.02 (1.74-5.24)** |  | 7.5 (151/2022) | 46.8 (52/111) | **8.98 (4.63-17.43)** |
| **By education level** |  |  |  |  |  |  |  |
| No previous education | 7.7 (110/1424) | 25.6 (11/43) | **6.19 (2.43-15.79)** |  | 7.1 (90/1260) | 46.5 (20/43) | **10.36 (3.75-28.65)** |
| Primary school | 6.0 (170/2857) | 33.0 (32/97) | **5.90 (3.10-11.21)** |  | 5.2 (173/3350) | 35.9 (46/128) | **8.18 (4.44-15.05)** |
| Middle school | 8.1 (232/2862) | 32.5 (37/114) | **4.83 (2.88-8.10)** |  | 4.5 (166/3697) | 25.6 (20/78) | **6.43 (3.06-13.50)** |
| College, University or above | 6.6 (156/2362) | 20.9 (28/134) | **2.97 (1.76-4.99)** |  | 6.2 (92/1493) | 31.1 (23/74) | **4.45 (2.06-9.61)** |
| **By occupation** |  |  |  |  |  |  |  |
| Unemployed | 10.3 (94/916) | 22.2 (8/36) | **3.34 (1.15-9.72)** |  | 6.8 (55/804) | 26.8 (11/51) | **3.88 (1.20-12.54)** |
| Farmer/herder | 5.8 (334/5744) | 36.5 (66/181) | **8.34 (5.32-13.06)** |  | 5.3 (372/7011) | 39.2 (87/222) | **8.23 (5.17-13.10)** |
| Civil servants/Personnel in public institutions | 8.1 (124/1537) | 22.8 (21/92) | **3.00 (1.63-5.51)** |  | 6.0 (63/1044) | 20.9 (9/43) | **4.74 (1.76-12.81)** |
| Other | 7.7 (138/1799) | 16.7 (18/108) | **2.59 (1.34-5.01)** |  | 5.1 (80/1560) | 30.0 (15/50) | **10.70 (3.93-29.14)** |
| **By religious belief** |  |  |  |  |  |  |  |
| No | 5.5 (216/3910) | 16.6 (26/157) | **2.81 (1.67-4.75)** |  | 4.1 (152/3728) | 23.4 (26/111) | **4.91 (2.51-9.59)** |
| Yes | 8.3 (434/5252) | 34.5 (79/229) | **5.20 (3.61-7.49)** |  | 6.4 (370/5755) | 44.7 (89/199) | **8.02 (5.15-12.51)** |
| **By monthly household income** |  |  |  |  |  |  |  |
| ≤ 3000 CNY | 7.1 (428/6050) | 28.6 (69/241) | **5.03 (3.40-7.43)** |  | 5.7 (354/6169) | 37.5 (81/216) | **5.71 (3.55-9.18)** |
| 3000 - 5000 CNY | 6.9 (162/2352) | 35.2 (32/91) | **5.36 (2.93-9.80)** |  | 4.7 (129/2754) | 29.1 (25/86) | **8.00 (3.33-19.23)** |
| > 5000 CNY | 6.4 (93/1462) | 14.1 (11/78) | 1.93 (0.90-4.17) |  | 5.8 (82/1424) | 28.8 (15/52) | **10.10 (4.55-22.43)** |
| **By significant negative life events** |  |  |  |  |  |  |  |
| No | 5.5 (495/9011) | 17.2 (53/308) | **3.53 (2.45-5.11)** |  | 3.4 (311/9231) | 13.5 (29/215) | **5.48 (3.26-9.19)** |
| Yes | 19.8 (195/985) | 55.7 (59/106) | **6.12 (3.49-10.72)** |  | 22.5(251/1115) | 67.1 (94/140) | **8.08 (4.60-14.22)** |
| **By chronic medical condition** |  |  |  |  |  |  |  |
| No | 6.2 (545/8832) | 24.4 (82/336) | **4.08 (2.93-5.67)** |  | 4.3 (380/8843) | 30.1 (84/279) | **8.09 (5.42-12.09)** |
| Yes | 14.6 (131/896) | 44.4 (28/63) | **5.98 (2.96-12.09)** |  | 12.2 (156/1276) | 47.6 (30/63) | **5.04 (2.18-11.67)** |

CNY, Chinese Yuan; PHQ-9, Patient Health Questionnaire-9

Appendix table 14: Gender-stratified associations between intimate partner violence victimization and **suicide attempt** by each characteristic (Qinghai, China. 2024)

|  | Female | | |  | Male | | |
| --- | --- | --- | --- | --- | --- | --- | --- |
|  | % (N_IPV_  / N_Overall)_ | PHQ-9 score ≥10 | |  | % (N_IPV_  / N_Overall)_ | PHQ-9 score ≥10 | |
|  |  | % (N_IPV_  / N_Overall)_ | OR (95% CI) |  |  | % (N_IPV_  / N_Overall)_ | OR (95% CI) |
| **By age groups (years)** |  |  |  |  |  |  |  |
| < 35 | 7.3 (295/4060) | 27.2 (31/114) | **4.04 (2.27-7.20)** |  | 5.6 (168/2982) | 35.2 (32/91) | **6.99 (3.08-15.87)** |
| 35-45 | 7.6 (198/2615) | 29.9 (20/67) | **5.11 (2.49-10.48)** |  | 4.5 (125/2761) | 26.6 (21/79) | **7.31 (3.43-15.59)** |
| 45-55 | 6.7 (119/1778) | 32.4 (12/37) | **7.09 (2.40-20.99)** |  | 5.6 (134/2393) | 39.6 (19/48) | **8.01 (2.96-21.69)** |
| 55 or more | 2.9 (32/1111) | 18.2 (2/11) | - |  | 4.9 (80/1642) | 36.8 (14/38) | **6.25 (2.09-18.72)** |
| **By living areas** |  |  |  |  |  |  |  |
| Pastoral area | 6.7 (437/6532) | 28.9 (56/194) | **4.79 (3.05-7.52)** |  | 5.5 (379/6934) | 34.2 (79/231) | **7.26 (4.47-11.80)** |
| Nonpastoral area | 6.7 (208/3101) | 22.5 (9/40) | **5.12 (2.20-11.92)** |  | 4.8 (142/2947) | 27.6 (8/29) | **4.67 (1.61-13.56)** |
| **By ethnic group** |  |  |  |  |  |  |  |
| Han | 5.6 (209/3704) | 19.2 (14/73) | **3.57 (1.76-7.23)** |  | 4.1 (146/3526) | 19.2 (14/73) | **4.31 (1.81-10.27)** |
| Others | 7.7 (370/4802) | 30.5 (36/118) | **5.91 (3.61-9.68)** |  | 5.8 (287/4987) | 35.0 (48/137) | **7.68 (4.51-13.09)** |
| **By marital status** |  |  |  |  |  |  |  |
| Married | 6.6 (491/7476) | 26.8 (42/157) | **5.21 (3.20-8.49)** |  | 4.7 (370/7813) | 30.8 (57/185) | **7.38 (4.47-12.19)** |
| Other | 7.3 (148/2031) | 30.1 (22/73) | **4.94 (2.36-10.36)** |  | 7.5 (138/1838) | 42.6 (29/68) | **4.94 (2.00-12.19)** |
| **By education level** |  |  |  |  |  |  |  |
| No previous education | 7.3 (98/1345) | 28.6 (8/28) | **9.89 (2.90-33.66)** |  | 6.5 (76/1176) | 47.1 (16/34) | **5.87 (1.89-18.22)** |
| Primary school | 5.8 (158/2733) | 25.8 (16/62) | **4.28 (1.82-10.03)** |  | 5.2 (162/3104) | 28.7 (31/108) | **6.02 (3.03-11.93)** |
| Middle school | 7.9 (213/2699) | 30.4 (21/69) | **5.27 (2.65-10.50)** |  | 4.3 (151/3481) | 32.7 (18/55) | **8.23 (3.45-19.62)** |
| College, University or above | 6.6 (150/2258) | 30.9 (17/55) | **4.06 (1.94-8.47)** |  | 6.2 (87/1414) | 41.9 (18/43) | **6.75 (2.20-20.74)** |
| **By occupation** |  |  |  |  |  |  |  |
| Unemployed | 10.6 (91/860) | 21.7 (5/23) | **5.58 (1.37-22.70)** |  | 7.0 (52/739) | 28.1 (9/32) | **4.83 (1.30-17.94)** |
| Farmer/herder | 5.4 (295/5427) | 31.0 (36/116) | **6.81 (3.80-12.20)** |  | 5.1 (333/6566) | 35.0 (63/180) | **7.17 (4.18-12.28)** |
| Civil servants/Personnel in public institutions | 7.9 (115/1457) | 33.3 (14/42) | **4.97 (2.24-11.02)** |  | 5.8 (56/961) | 25.0 (4/16) | 4.67 (0.77-28.13) |
| Other | 7.9 (136/1732) | 18.8 (9/48) | **3.08 (1.19-7.98)** |  | 5.0 (73/1457) | 36.7 (11/30) | **10.53 (3.31-33.46)** |
| **By religious belief** |  |  |  |  |  |  |  |
| No | 5.4 (202/3718) | 19.2 (15/78) | **2.98 (1.36-6.54)** |  | 4.1 (143/3506) | 27.5 (22/80) | **6.08 (2.56-14.46)** |
| Yes | 8.1 (403/5006) | 34.8 (47/135) | **6.11 (3.81-9.81)** |  | 6.2 (338/5425) | 41.1 (58/141) | **6.91 (4.11-11.62)** |
| **By monthly household income** |  |  |  |  |  |  |  |
| ≤ 3000 CNY | 6.8 (391/5732) | 24.6 (35/142) | **5.16 (3.04-8.78)** |  | 5.4 (312/5740) | 35.0 (56/160) | **4.86 (2.73-8.63)** |
| 3000 - 5000 CNY | 6.9 (154/2234) | 44.2 (23/52) | **8.63 (3.71-20.11)** |  | 4.7 (123/2595) | 34.9 (22/63) | **9.56 (3.47-26.31)** |
| > 5000 CNY | 6.2 (86/1388) | 17.1 (6/35) | 2.24 (0.79-6.33) |  | 5.4 (72/1323) | 27.6 (8/29) | **15.60 (5.26-46.29)** |
| **By significant negative life events** |  |  |  |  |  |  |  |
| No | 5.4 (465/8556) | 18.7 (31/166) | **4.32 (2.66-7.00)** |  | 3.3 (282/8631) | 12.7 (20/157) | **4.70 (2.45-9.01)** |
| Yes | 19.0 (176/924) | 52.4 (33/63) | **7.60 (3.29-17.55)** |  | 22.1 (230/1041) | 67.7 (67/99) | **9.45 (4.68-19.07)** |
| **By chronic medical condition** |  |  |  |  |  |  |  |
| No | 6.1 (508/8391) | 24.3 (45/185) | **4.81 (3.09-7.48)** |  | 4.1 (341/8261) | 29.2 (59/202) | **7.86 (4.84-12.78)** |
| Yes | 14.5 (124/856) | 54.5 (18/33) | **7.14 (2.67-19.07)** |  | 12.4 (150/1211) | 46.7 (21/45) | **4.82 (1.81-12.81)** |

CNY, Chinese Yuan; PHQ-9, Patient Health Questionnaire-9
